# Evaluation of carbapenem inactivation method-based phenotypic assays for the detection of GES-type carbapenemases in Enterobacterales, *Pseudomonas aeruginosa*, and *Acinetobacter baumannii*

**DOI:** 10.1128/jcm.00737-26

**Published:** 2026-08-21

**Authors:** Samuel García-García, Jorge Rodríguez-Grande, María Siller-Ruíz, Cristina Arjona-Torres, Esther Viedma, María Díez-Aguilar, Marta Hernández-García, Javier Fernández-Domínguez, Emilia Cercenado, Fernando Lázaro-Perona, Jesús Oteo-Iglesias, Patricia Ruiz-Garbajosa, Rafael Cantón, Nuria Valcárcel-Fraile, Lucía Gallego, María Pía Roiz-Mesones, Jorge Calvo-Montes, Luis Martínez-Martínez, Alain Ocampo-Sosa, Sergio García-Fernández, on behalf of the GEMARA-GESPABE Study Group

**Affiliations:** 1 Servicio de Microbiología, Hospital Universitario Marqués de Valdecilla – IDIVAL, Santander, Spain; 2 CIBER de Enfermedades Infecciosas-CIBERINFEC, Instituto de Salud Carlos III 38176, Madrid, Spain; 3 Instituto Maimónides de Investigación Biomédica de Córdoba 215147, Córdoba, Spain; 4 Servicio de Microbiología, Hospital Universitario 12 de Octubre, Instituto de Investigación Hospital 12 de Octubre (Imas 12) 542148, Madrid, Spain; 5 Servicio de Microbiología, Hospital Universitario La Princesa, Madrid, Spain; 6 Hospital Universitario Ramón y Cajal-IRYCIS 537482 , Madrid, Spain; 7 Servicio de Microbiología, Hospital Universitario Central de Asturias 16474, Oviedo, Spain; 8 Servicio de Microbiología, Hospital General Universitario Gregorio Marañón 16483, Madrid, Spain; 9 CIBER de Enfermedades Respiratorias-CIBERES, Instituto de Salud Carlos III 38176, Madrid, Spain; 10 Servicio de Microbiología, Hospital Universitario La Paz 16268, Madrid, Spain; 11 Laboratorio de Resistencia a Antibióticos, Centro Nacional de Microbiología, Instituto de Salud Carlos III 38176, Madrid, Spain; 12 Laboratorio de Antibióticos y Bacteriología Molecular, Departamento de Inmunología, Microbiología y Parasitología, Facultad de Medicina y Enfermería, Universidad del País Vasco 58349, Leioa, Spain; 13 UGC de Microbiología, Hospital Universitario Reina Sofía 16501, Córdoba, Spain; 14 Departamento de Química Agrícola, Edafología y Microbiología, Universidad de Córdoba 16735, Córdoba, Spain; 15 Departamento de Biología Molecular, Universidad de Cantabria 16761, Santander, Spain; Mayo Clinic, Rochester, Minnesota, USA

**Keywords:** carbapenem inactivation method, GES, carbapenemases

## Abstract

Detection of GES-type carbapenemases remains challenging because of their low prevalence and frequently weak hydrolytic activity against carbapenems. Carbapenem inactivation method (CIM)-based assays are widely used as phenotypic screening tools for carbapenemase detection; however, their performance in large collections of GES producers has not been systematically evaluated. We assessed the performance of CIM, modified CIM (mCIM), and CIM-Tris in a diverse collection of GES-producing clinical isolates, including 110 Enterobacterales and 108 *Pseudomonas aeruginosa,* recovered from Spanish hospitals (2010–2024), and 10 *Acinetobacter baumannii* isolates, mostly obtained from a hospital in Egypt. Whole-genome sequencing was carried out for species confirmation and resistome analysis. Meropenem MICs were determined by broth microdilution. Overall, 92.1% of isolates were GES-carbapenemase producers (CP), whereas 7.9% expressed GES-type extended-spectrum β-lactamases (ESBLs). In Enterobacterales (predominantly carrying *bla*_GES-6_), mCIM improved sensitivity compared with CIM (63.6% vs 40.0%), although many isolates remained undetected due to low meropenem MICs (MIC_50_, 0.5 µg/mL). In CP-*P. aeruginosa* (mainly *bla*_GES-5_), CIM, mCIM, and CIM-Tris showed sensitivities of 89.1%, 94.6%, and 100%, respectively; however, CIM-Tris yielded false-positive results in 50% of non-CP isolates (mostly *bla*_GES-1_ producers). Meropenem MICs in *P. aeruginosa* were higher (MIC_50_, >32 µg/mL). In *A. baumannii*, CIM-Tris improved sensitivity compared with CIM (100% vs 25.0%). These findings indicate that CIM-based methods can detect GES-type carbapenemases, but performance varies according to bacterial species and GES variant, and reduced specificity may occur in isolates producing GES-type ESBLs. Complementary molecular testing may therefore be necessary to ensure accurate detection of GES-type carbapenemases in routine clinical laboratories.

**IMPORTANCE:**

GES-type carbapenemases represent an important but underrecognized diagnostic challenge due to their low global prevalence, heterogeneous hydrolytic activity, and the limited performance data available for routine phenotypic detection methods. Although CIM-based assays are widely implemented in clinical microbiology laboratories for carbapenemase screening, their performance against GES-producing organisms has not been comprehensively evaluated across different bacterial genera and GES variants. In this study, we evaluated the performance of CIM, modified CIM (mCIM), and CIM-Tris in a large multicenter collection of well-characterized GES-producing clinical isolates, including Enterobacterales, *Pseudomonas aeruginosa*, and *Acinetobacter baumannii*. Our findings demonstrate substantial variability in assay performance according to bacterial species and GES variant. Notably, mCIM improved sensitivity among Enterobacterales with low meropenem MICs, whereas CIM-Tris achieved excellent sensitivity in *P. aeruginosa* and *A. baumannii* but at the expense of reduced specificity in isolates producing GES-type ESBLs. To the best of our knowledge, this is the first study directly comparing multiple CIM-based approaches in such a large and taxonomically diverse collection of GES-producing isolates.

## INTRODUCTION

The World Health Organization (WHO) declared antimicrobial resistance (AMR) as one of the most urgent threats to global health, with major consequences for patient outcomes in healthcare settings (1). Recent analyses estimate that bacterial AMR was associated with 4.71 million deaths worldwide in 2021 (2). In particular, carbapenem-resistant Gram-negative bacterial infections in hospitalized patients have increased more rapidly than for infections resistant to any other antibiotic class, contributing to more than a million deaths in 2021 (2). In the 2024 WHO Bacterial Priority Pathogens List, carbapenem-resistant *Acinetobacter baumannii* Enterobacterales and *Pseudomonas aeruginosa* were classified as pathogens of critical or high priority (1). Therefore, prompt and accurate detection of carbapenemase producers is critical for infection control and for ensuring appropriate therapy (3).

The most common carbapenem-resistance mechanisms involve carbapenemases, such as KPC, OXA-48-like, NDM, VIM, and IMP, which are widely disseminated and can be accurately detected using a variety of diagnostic techniques. The main challenge arises with less common carbapenemases, such as GES (Guiana extended-spectrum), IMI, GIM, and other infrequent enzymes, which are rarely encountered in routine practice. The GES β-lactamase family is a representative example of this problem (4). Initially, GES-1 was described as an extended-spectrum β-lactamase (ESBL), capable of hydrolyzing penicillins and cephalosporins (5). Over time, new variants emerged with mutations in the enzyme active site that expanded their substrate profile, and some of them, such as GES-5 and GES-6, developed carbapenemase activity (6–8). Detection of these carbapenemase-active variants remains difficult because of the limited availability of commercial assays and often weak hydrolytic activity against carbapenems, which may lead to underdiagnosis and an underestimation of their prevalence (9). However, GES-type carbapenemases, together with VIM, are among the most frequently identified carbapenemases in *P. aeruginosa* in different surveillance studies (10, 11).

One of the most commonly used phenotypic methods for carbapenemase screening is the carbapenem inactivation method (CIM) (12). This method allows for a rapid response regarding the presence of a carbapenemase in a suspect isolate, which is key to optimizing antibiotic treatment and initiating prevention measures. Although multiple studies have demonstrated the effectiveness of CIM and its variants for detecting the “big five carbapenemases,” less common carbapenemases, including GES-type enzymes, have been underrepresented in most evaluation studies (13–15).

The objectives of this study were (i) to assess the sensitivity of CIM and modified CIM (mCIM) in Enterobacterales, (ii) to assess the sensitivity of CIM, mCIM, and modified CIM using Tris-HCl buffer (CIM-Tris) in *P. aeruginosa,* and (iii) to assess the sensitivity of CIM and CIM-Tris in *A. baumannii*, using a collection of Gram-negative bacilli carrying GES-type β-lactamases.

## MATERIALS AND METHODS

### Bacterial isolates

As part of a retrospective multicenter study promoted by the Study Group on Mechanisms of Action and Resistance to Antimicrobials (GEMARA) of the Spanish Society of Infectious Diseases and Clinical Microbiology (SEIMC), involving 14 hospitals across Spain, a total of 110 Enterobacterales and 92 *P. aeruginosa* isolates producing GES-type carbapenemases collected between 2010 and 2024 were included. A further 16 *P. aeruginosa* strains producing GES-type ESBLs were included as negative controls. Additionally, 10 GES-producing *A. baumannii* isolates were included in the study: nine from a hospital in Alexandria, Egypt, and one from Spain. All *A. baumannii* isolates harbored GES-type ESBLs and co-produced additional oxacillinases. Only one isolate per patient was included across the entire study collection.

### Identification and antimicrobial susceptibility testing

Species identification was performed using the MALDI-TOF VITEK MS Prime (bioMérieux, France), according to the manufacturer’s instructions. MICs of ertapenem, imipenem, and meropenem were determined by ISO standardized broth microdilution in all isolates included in the study (16). MIC results were interpreted in accordance with the current v-16.0 European Committee on Antimicrobial Susceptibility Testing (EUCAST) clinical breakpoints (17). *Escherichia coli* ATCC 25922, *Klebsiella pneumoniae* ATCC 700603 and *P. aeruginosa* ATCC 27853 were included as quality control strains.

### Whole-genome sequencing

Whole-genome sequencing (WGS) was performed for all isolates included in the study. DNA was extracted from Enterobacterales and *A. baumannii* isolates with the DNeasy Blood and Tissue Extraction Kit (Qiagen, Hilden, Germany), whereas DNA from *P. aeruginosa* was isolated using the DNeasy PowerSoil Pro Kit (Qiagen, Hilden, Germany). DNA concentration was measured in a Qubit 4 Fluorometer along with the 1x dsDNA HS Assay Kit (Thermo Fisher Scientific, Wilmington, DE, USA), following the manufacturer’s instructions. Libraries were prepared with the Rapid Barcoding Kit SQK-RBK117.96, sequenced on the MinION Mk1B platform with R10.4.1 FlowCells (Oxford Nanopore Technologies), and raw reads produced using the super-accurate (SUP) base-calling model. Raw reads were preprocessed with chopper v.0.7.0 (18), which required a minimum Phred average quality of 12, a minimum length of 300, and trimming the first 20 nucleotides of the reads. Genomes were assembled with Flye v.2.9.3-b1797 (19) and polished with Medaka v.1.11.3 model r941_e81_sup_g514 (Oxford Nanopore Technologies Ltd.). Contamination was ruled out using Kraken2 on raw reads (20) and Gambit taxonomic assignment on complete genomes (21). Antimicrobial resistance genes were annotated with Abricate v.1.0.1 (22) using the NCBI AMRFinderPlus database (23).

### Carbapenemase detection assays (CIM, mCIM, and CIM-Tris)

**Fig 1:**
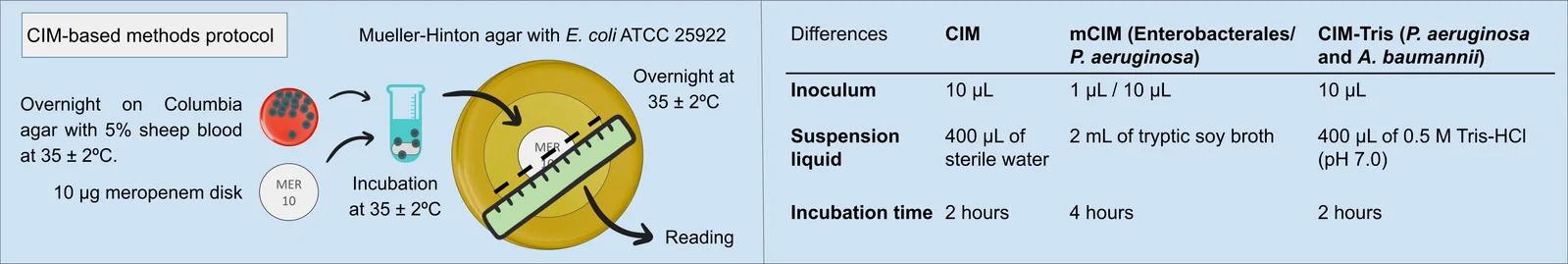
Procedural workflow for CIM-variants and technical protocol specifications.

The three CIM-variant methodologies employed in the study are summarized in Fig. 1.

The standard CIM was performed according to the original protocol described by van der Zwaluw (12). Bacterial isolates were grown overnight on Columbia agar with 5% sheep blood (bioMérieux, France) at 35°C ± 2°C. A full 10 μL inoculation loop of bacterial culture was suspended in 400 μL of sterile water, and a 10 μg meropenem disk (Oxoid, Basingstoke, UK) was added to the suspension. The incubation time was 2 h at 35°C ± 2°C.

For Enterobacterales and *P. aeruginosa*, the mCIM was performed following the procedure described by CLSI M100 (24). Briefly, 1 μL (Enterobacterales) or 10 μL (*P. aeruginosa*) loopful of test strain was emulsified in 2 mL of tryptic soy broth. A 10 μg meropenem disk was immersed in the suspension and incubated for 4 h at 35°C ± 2°C.

CIM-Tris was applied for *P. aeruginosa* and *A. baumannii* isolates. In this method, a 10 μL loop of bacterial colonies was suspended in 400 μL of 0.5 M Tris-HCl (pH 7.0), and a 10 μg meropenem disk was added. A 2-h incubation at 35°C ± 2°C was performed.

After the incubation periods, each disk was lightly pressed to remove excess moisture and then placed onto a Mueller-Hinton agar plate previously inoculated with *E. coli* ATCC 25922 as the susceptible indicator strain. Plates were incubated overnight at 35°C ± 2°C, and carbapenemase activity was determined according to the size of the inhibition zone around the disk. In all three methods, carbapenemase production was considered positive for isolates showing an inhibition zone of 6–15 mm or a zone of 16–18 mm with colonies within the inhibition zone. An inhibition zone ≥19 mm was classified as carbapenemase negative. Zones of 16–18 mm without internal colonies were recorded as indeterminate. The cut-off values applied in this study corresponded to those established by CLSI M100-S27 for the mCIM and to those described in the original publication of the CIM-Tris method (24, 25). As the original CIM protocol does not define cut-off criteria, the same thresholds used for mCIM and CIM-Tris were applied to enable comparative sensitivity assessments.

Investigators were blinded to genus, species, and GES-variant status when reading the phenotypic tests. Sensitivity was determined by using WGS as the gold standard.

## RESULTS

A total of 228 non-duplicate clinical isolates of GES β-lactamase producers (Enterobacterales, *n* = 110; *P. aeruginosa*, *n* = 108; and *A. baumannii*, *n* = 10) were collected (Table 1). All Enterobacterales and 92 *P. aeruginosa* isolates produced exclusively GES-type carbapenemases, whereas the further 16 *P. aeruginosa* and all *A. baumannii* strains produced GES-type ESBLs. These isolates were collected from 14 hospitals in Spain (*n* = 219) and Egypt (*n* = 9), over a period spanning from 2010 to 2024. Detailed isolate characteristics (species, location, year of isolation, source, sequence type, resistance mechanisms, and ertapenem, imipenem, and meropenem MIC) are provided in Table S1. The main findings of the study are summarized in Tables 2 and 3.

**TABLE 1:** Final bacterial isolate collection of GES-β-lactamase producers included in the evaluation performance of CIM-based methods

	*E. cloacae* complex	*Klebsiella* spp.^*a*^	*S. marcescens*	Other Enterobacterales^*b*^	*P. aeruginosa*	*A. baumannii*	Subtotal
Carbapenemase producers							
GES-1^*c*^+GES-5^*d*^					2		2
GES-5^*d*^					87		87
GES-6^*d*^	79	15	10	3			107
GES-11^*c*^+OXA-23						1	1
GES-11^*c*^+OXA-23+OXA-94^*e*^						3	3
GES-14^*d*^		1					1
GES-19^*c*^+GES-20^*d*^					3		3
GES-35^*c*^+OXA-23+OXA-65^*e*^						4	4
GES-53^*d*^	1						1
GES-55^*d*^			1				1
Non-carbapenemase producers							
GES-1^*c*^					15		15
GES-15^*c*^					1		1
GES-35^*c*^+OXA-65^*e*^						2	2
Total number	80	16	11	3	108	10	228

^*a*^ Species in *Klebsiella* spp. included *K. oxytoca* (*n* = 10), *K. pneumoniae* (*n* = 4), *K. variicola* (*n* = 1), *K. michiganensis* (*n* = 1).

^*b*^ Species in other Enterobacterales included *Citrobacter freundii* complex (*n* = 1), *Citrobacter amalonaticus* (*n* = 1), and *Escherichia hermannii* (*n* = 1).

^*c*^ GES enzymes with ESBL activity.

^*d*^ GES enzymes with carbapenemase activity.

^*e*^ OXA-type β-lactamase encoding genes that are intrinsic to *A. baumannii*.

**TABLE 2:** CIM, mCIM, and CIM-Tris method performances in a collection of GES-β-lactamase producers^*i*^

Species (*n*)	CIM		mCIM		CIM-Tris	
	Sens % (CI)	Ind %	Sens % (CI)	Ind %	Sens % (CI)	Ind %
Carbapenemase producers						
Enterobacterales (110)	40.0 (30.8–49.2)	0.9	63.6 (54.6–72.6)	4.6	–	–
*E. cloacae* complex^*a*^ (80)	23.8 (14.4–33.1)	0	52.5 (41.6–63.4)	6.3	–	–
*Klebsiella* spp.^*b*^ (16)	93.8 (81.9–100)	0	93.8 (81.9–100)	0	–	–
*S. marcescens*^*c*^ (11)	81.8 (59.0–100)	9.1	100 (74.1–100)	0	–	–
Other Enterobacterales^*d*^ (3)	33.3 (0–86.6)	0	66.6 (13.8–100)	0	–	–
*P. aeruginosa*^*e*^ (92)	89.1 (82.8–95.5)	1.1	94.6 (89.9–99.2)	1.1	100 (96.0–100)	0
*A. baumannii*^*f*^ (8)	25.0 (0–55.0)	0	–	–	100 (63.1–100)	0
Non-carbapenemase producers						
*P. aeruginosa*^*g*^ (16)	0 (0–19.4)	0	0 (0–19.4)	0	50.0 (28.0–72.0)	18.8
*A. baumannii*^*h*^ (2)	0 (0–84.2)	0	–	–	50.0 (1.3–98.7)	0

^*a*^ GES variants in *E. cloacae* complex included *bla*_GES-6_ (98.8%) and *bla*_GES-53_ (1.2%).

^*b*^ Species in *Klebsiella* spp. included *K. oxytoca* (*n* = 10), *K. pneumoniae* (*n* = 4), *K. variicola* (*n* = 1), *K. michiganensis* (*n* = 1). GES variants in *Klebsiella* spp. included *bla*_GES-6_ (93.8%) and *bla*_GES-14_ (6.2%).

^*c*^ GES variants in *S. marcescens* included *bla*_GES-6_ (90.9%) and *bla*_GES-55_ (9.1%).

^*d*^ Species in other Enterobacterales included *Citrobacter freundii* complex (*n* = 1), *Citrobacter amalonaticus* (*n* = 1), *Escherichia hermannii* (*n* = 1). GES variants in other Enterobacterales included *bla*_GES-6_ (100%).

^*e*^ GES variants in *P. aeruginosa* carbapenemase producers included *bla*_GES-5_ (94.6%), *bla*_GES-19+GES-20_ (3.3%), and *bla*_GES-1+GES-5_ (2.3%).

^*f*^ GES variants in *A. baumannii* carbapenemase producers included *bla*_GES-35_+*bla*_OXA-23_*+bla*_OXA-65_ (50.0%), *bla*_GES-11_+*bla*_OXA-23_*+bla*_OXA-94_ (37.5%), and *bla*_GES-11_+*bla*_OXA-23_ (12.5%).

^*g*^ GES variants in *P. aeruginosa* non-carbapenemase producers included *bla*_GES-1_ (93.8%) and *bla*_GES-15_ (6.3%).

^*h*^ GES variants in *A. baumannii* non-carbapenemase producers included *bla*_GES-35_+*bla*_OXA-65_ (100.0%).

^*i*^ Sens, sensitivity; CI, confidence interval; Ind, indeterminate; –, not tested.

**TABLE 3:** Activity of ertapenem, imipenem, and meropenem in a collection of GES-β-lactamase producers^*i*^

Species (*n*)	Ertapenem						Imipenem					Meropenem					
	Range (µg/mL)	MIC_50/90_ (µg/mL)	S (%)	I (%)	R (%)	SC (%)	Range (µg/mL)	MIC_50/90_ (µg/mL)	S (%)	I (%)	R (%)	Range (µg/mL)	MIC_50/90_ (µg/mL)	S (%)	I (%)	R (%)	SC (%)
Carbapenemase producers																	
Enterobacterales (110)	0.12 to >32	2/32	21.8	0	78.2	99.1	0.25 to >32	0.5/4	90.0	3.6	6.4	0.06–32	0.5/4	90.0	0	10.0	75.5
*E. cloacae* complex^*a*^ (80)	0.12 to >32	2/8	21.2	0	78.8	98.8	0.25 to >32	0.5/1	100	0	0	0.06–32	0.5/2	100	0	0	76.3
*Klebsiella* spp.^*b*^ (16)	0.25 to >32	1/32	31.3	0	68.7	100	0.25–16	0.5/2	93.7	0	6.3	0.06–32	0.25/1	93.7	0	6.3	56.3
*S. marcescens*^*c*^ (11)	16 to >32	32/>32	0	0	100	100	0.5–8	8/8	9.1	36.4	54.5	1–32	16/16	9.1	0	90.9	100
Other Enterobacterales^*d*^ (3)	0.25–4	0.5/4	66.6	0	33.3	66.6	0.25–1	0.5/1	100	0	0	0.06–1	0.25/1	100	0	0	66.6
*P. aeruginosa*^*e*^ (92)	–	–	–	–	–	–	16 to >32	32/32	0	0	100	32 to >32	>32/>32	0	0	100	–
*A. baumannii*^*f*^ (8)	–	–	–	–	–	–	16–32	16/32	0	0	100	16–32	32/>32	0	0	100	–
Non-carbapenemase producers																	
*P. aeruginosa*^*g*^ (16)	–	–	–	–	–	–	4–16	8/16	0	25.0	75.0	2–32	8/32	6.2	75.0	18.8	–
*A. baumannii*^*h*^ (2)	–	–	–	–	–	–	2–2	2/2	100	0	0	2 to >32	2/2	100	0	0	–

^*a*^ GES variants in *E. cloacae* complex included *bla*_GES-6_ (98.8%) and *bla*_GES-53_ (1.2%).

^*b*^ Species in *Klebsiella* spp. included *K. oxytoca* (*n* = 10), *K. pneumoniae* (*n* = 4), *K. variicola* (*n* = 1), and *K. michiganensis* (*n* = 1). GES variants in *Klebsiella* spp. included *bla*_GES-6_ (93.8%) and *bla*_GES-14_ (6.2%).

^*c*^ GES variants in *S. marcescens* included *bla*_GES-6_ (90.9%) and *bla*_GES-55_ (9.1%).

^*d*^ Species in other Enterobacterales included *Citrobacter freundii* complex (*n* = 1), *Citrobacter amalonaticus* (*n* = 1), and *Escherichia hermannii* (*n* = 1). GES variants in other Enterobacterales included *bla*_GES-6_ (100%).

^*e*^ GES variants in *P. aeruginosa* carbapenemase producers included *bla*_GES-5_ (94.6%), *bla*_GES-19+GES-20_ (3.3%), and *bla*_GES-1+GES-5_ (2.3%).

^*f*^ GES variants in *A. baumannii* carbapenemase producers included *bla*_GES-35_+*bla*_OXA-23_*+bla*_OXA-65_ (50.0%), *bla*_GES-11_+*bla*_OXA-23_*+bla*_OXA-94_ (37.5%), and *bla*_GES-11_+*bla*_OXA-23_ (12.5%).

^*g*^ GES variants in *P. aeruginosa* non-carbapenemase producers included *bla*_GES-1_ (93.8%) and *bla*_GES-15_ (6.3%).

^*h*^ GES variants in *A. baumannii* non-carbapenemase producers included *bla*_GES-35_+*bla*_OXA-65_ (100.0%).

^*i*^ S, susceptible; I, susceptible increased exposure; R, resistance; SC, positive carbapenemase screening cut-off; –, not tested.

### Enterobacterales

Overall, 97.3% of Enterobacterales isolates carried the *bla*_GES-6_ carbapenemase variant. Despite this, meropenem retained substantial activity against this group (90.0%; MIC_50_, 0.5 µg/mL), although its activity varied across species, ranging from 100% susceptibility in *Enterobacter cloacae* complex to 9.1% in *Serratia marcescens*. Meropenem-resistant isolates included one *K. pneumoniae* (*bla*_GES-6_) and 10 *S. marcescens* (*bla*_GES-6_). The only meropenem-susceptible *S. marcescens* carried a *bla*_GES-55_ variant, which has been described as conferring resistance to ceftazidime/avibactam and collateral susceptibility to meropenem (26). Overall, these isolates exhibited similar and reduced susceptibility to imipenem (90.0%; MIC_50_, 0.5 µg/mL) and ertapenem (21.8%; MIC_50_, 2 µg/mL), respectively. Interestingly, 24.5% (27/110) and 0.9% (1/110) (*E. cloacae* complex isolate carrying *bla*_GES-6_) of isolates had meropenem and ertapenem MICs below the EUCAST screening cut-off for carbapenemase-producing Enterobacterales (>0.12 µg/mL), respectively (27).

**Fig 2:**
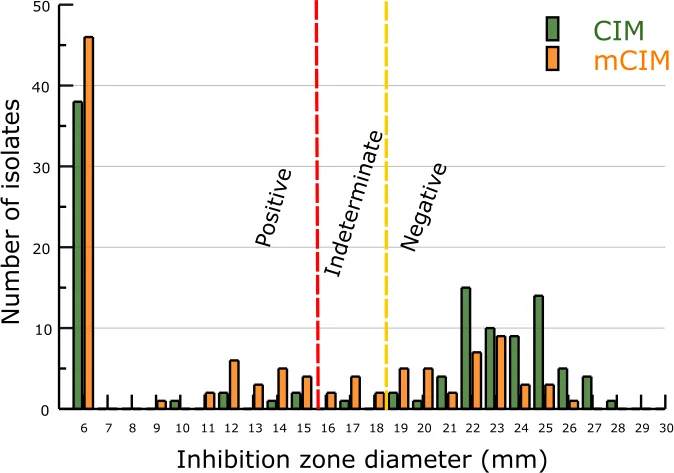
Distribution of the CIM (green) and mCIM (orange) zone diameter measurements for GES-type carbapenemase-producing Enterobacterales. Red and yellow vertical dashed lines demarcate the thresholds for positive (<16 mm), indeterminate (16–18 mm), and negative (>18 mm) CIM and mCIM results, respectively.

**Fig 3:**
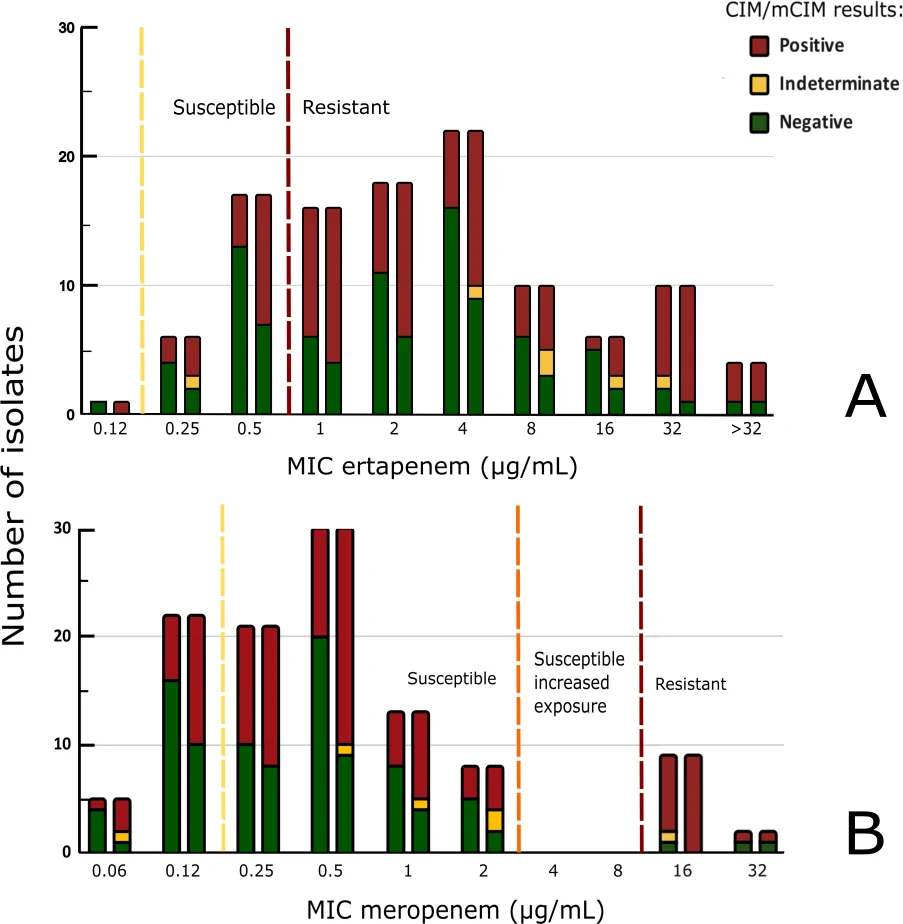
(**A**) Distribution of CIM results (first column) and mCIM results (second column) according to ertapenem MIC in Enterobacterales. Red vertical dashed lines demarcate the breakpoints for susceptible (<1 µg/mL) and resistant (≥1 µg/mL) ertapenem MIC for Enterobacterales, respectively. (**B**) Distribution of CIM results (first column) and mCIM results (second column) according to meropenem MIC in Enterobacterales. Orange and red vertical dashed lines demarcate the breakpoints for susceptible (<4 µg/mL), increased exposure susceptible (4–8 µg/mL), and resistant (>8 µg/mL) meropenem MIC for Enterobacterales, respectively. CIM and mCIM results are displayed as negative (green), indeterminate (yellow), and positive (red). The yellow vertical dashed line shows the EUCAST meropenem and ertapenem screening cut-off for carbapenemase-producing Enterobacterales (>0.12 µg/mL).

Among the 110 Enterobacterales isolates, CIM sensitivity was 40.0%, with marked species-specific variation (93.8% in *Klebsiella* spp., 81.8% in *S. marcescens*, and 23.8% in the *E. cloacae* complex). mCIM increased overall sensitivity to 63.6% (+23.6% compared with CIM), reaching 52.5% sensitivity in *E. cloacae* complex and 93.8% and 100% in *Klebsiella* spp. and *S. marcescens*, respectively. Inhibition zone diameters are shown in Fig. 2; compared with CIM, mCIM yielded significantly smaller zones, particularly in isolates with a meropenem MIC of 0.5 µg/mL (Fig. 3). Interestingly, among the 24 isolates susceptible to all three carbapenems, mCIM improved sensitivity by 33.3% compared with CIM.

### P. aeruginosa

Against *P. aeruginosa*, meropenem displayed distinct activity profiles between the 92 GES producers with carbapenemase activity (CP) and the 16 GES producers with non-carbapenemase activity (NCP) isolates. While CP isolates, mostly producing *bla*_GES-5_ variant, were uniformly resistant to meropenem (MIC_50_, >32 µg/mL) and imipenem (MIC_50_, 32 µg/mL), NCP isolates, predominantly carrying *bla*_GES-1_, displayed substantially lower MICs (MIC_50_, 8 µg/mL for both carbapenems) and higher susceptibility rates.

Among CP-*P. aeruginosa* isolates, the sensitivities of CIM and mCIM were 89.1% and 94.6%, respectively, and all positive results showed an inhibition zone diameter of 6 mm. According to GES-variant, CIM and mCIM sensitivities were 89.9% and 94.4%, respectively, in isolates producing *bla*_GES-5_ alone or *bla*_GES-5_+*bla*_GES-1_, whereas for isolates carrying *bla*_GES-19_+*bla*_GES-20_, sensitivity was 66.6% with CIM and 100% with mCIM. CIM-Tris increased sensitivity to 100% for detection of CP-*P. aeruginosa*, with all isolates displaying a 6-mm inhibition zone. In contrast, among NCP isolates, CIM and mCIM yielded no positive results, whereas CIM-Tris produced false-positive results in 50.0% of cases, with a mean inhibition zone of 10.5 mm, and indeterminate results in an additional 18.8%.

### A. baumannii

*A. baumannii* isolates also showed marked differences in meropenem susceptibility according to their β-lactamase profile. All isolates harbored GES-type ESBL genes (*bla*_GES-11_ or *bla*_GES-35_) together with additional *bla*_OXA-51_-like genes (*bla*_OXA-65_ or *bla*_OXA-94_) and *bla*_ADC_ genes (*bla*_ADC-25_, *bla*_ADC-73_, *bla*_ADC-80_, and *bla*_ADC-117_). Additionally, the 80.0% co-expressed *bla*_OXA-23_, exhibiting higher-level resistance to meropenem (MIC_50_, 32 µg/mL) and imipenem (MIC_50_, 16 µg/mL). In contrast, the remaining 20%, which carried *bla*_GES-35_ and *bla*_OXA-65_, displayed considerably lower meropenem and imipenem MICs (2 µg/mL). Among OXA-23-producing isolates, CIM sensitivity was 25.0% with a mean inhibition zone diameter of 19.3 mm. The positive isolates showed inhibition zones of 15 and 17 mm with intrazone colonies, respectively. CIM-Tris improved detection to 100% (+75.0%), with all isolates displaying a 6-mm inhibition zone. The two remaining non-carbapenemase-producing isolates (*bla*_GES-35_+*bla*_OXA-65_) exhibited negative CIM with an inhibition zone diameter of 25 mm. However, CIM-Tris yielded one false-positive result with an inhibition zone diameter of 6 mm.

## DISCUSSION

In this study, we evaluated the performance of CIM-based phenotypic assays for detecting GES-type carbapenemases in a diverse collection of Enterobacterales, *P. aeruginosa*, and *A. baumannii*. We found that the diagnostic performance of CIM, mCIM, and CIM-Tris varied markedly according to bacterial genus and GES variants, reflecting fundamental differences in carbapenem hydrolytic activity and resistance phenotypes. Overall, no single CIM-based approach demonstrated optimal performance across all species; rather, each method showed distinct strengths and limitations depending on the epidemiological and microbiological context.

Molecular methods are widely used for carbapenemase detection, whereas phenotypic assays, such as CIM, offer notable advantages, including low cost and the potential to detect carbapenemase activity regardless of the underlying molecular mechanism (28). Furthermore, these methods have a real impact, since detecting carbapenemases is crucial for guiding antibiotic treatment to improve patient prognosis, as well as for initiating prevention measures that reduce costs derived from the spread of resistance (29). mCIM is currently recommended by CLSI for Enterobacterales and *P. aeruginosa*, but not for *Acinetobacter* spp. due to its poor sensitivity in that genus (24). In meta-analyses focused mainly on common carbapenemases, mCIM has shown excellent performance (29). However, data on less prevalent enzymes, such as GES-type carbapenemases, remain limited, particularly across different bacterial genera.

In Enterobacterales, our results are consistent with previous reports showing that *bla*_GES-6_-producing isolates often display low meropenem MICs, reflecting the low hydrolytic activity of this enzyme against carbapenems (30). Consequently, these carbapenemases may remain undetected when meropenem MICs fall below the screening cut-off for carbapenemase-producing Enterobacterales (>0.12 µg/mL) (27). In our collection, 24.5% of Enterobacterales isolates fell below this cut-off. The low meropenem MICs explain the poor sensitivity of the standard CIM in *E. cloacae* complex, which has also been described by other authors (31). This situation highlights the risk of misclassifying these isolates as ESBL or AmpC hyperproducers, which could lead to the clinical use of meropenem instead of more suitable options for class A carbapenemases, such as ceftazidime-avibactam or meropenem-vaborbactam. Given the elevated ertapenem MICs observed in this group, using ertapenem along with meropenem for carbapenemase screening may represent a sensitive strategy. Indeed, incorporating an ertapenem disk into the CIM could improve GES detection, as this agent appears to be more susceptible to hydrolysis than meropenem within our collection. However, further studies are necessary to formally evaluate the performance of this modification and the potential loss of specificity. Regardless of carbapenem MIC values, mCIM significantly improves sensitivity independently, in line with previous studies showing enhanced carbapenem hydrolysis detection with this modified protocol (32–35). The enhanced sensitivity of the mCIM among carbapenem-susceptible isolates is particularly interesting, highlighting the potential for harboring carbapenemases even in strains with low MICs. These findings highlight the limitations of relying solely on MIC-based interpretation and reinforce current CLSI and EUCAST recommendations advocating for characterization of carbapenemase mechanisms alongside susceptibility testing, which is particularly relevant for guiding appropriate antimicrobial therapy (17, 24).

In *P. aeruginosa*, we observed a clear distinction between CP isolates, predominantly carrying *bla*_GES-5_, and NCP isolates, mainly harboring *bla*_GES-1_. The high sensitivity of CIM (89.1%) and especially of mCIM (94.6%) observed among GES-5-producing isolates is consistent with the greater hydrolytic activity of this variant (8, 36), whereas the absence of CIM positivity in GES-1-producing isolates supports the good specificity of both methods. Although CIM-Tris further increased sensitivity, this came at the cost of a marked reduction in specificity, a finding that aligns with previous reports describing instability of meropenem in Tris-HCl-based media and an increased risk of false-positive results (28, 37).

In *A. baumannii*, detection of GES-type β-lactamases remains particularly challenging due to the frequent co-production of class D oxacillinases, especially *bla*_OXA-23_ (38). The isolates harboring *bla*_OXA-23_ correlated with elevated meropenem MICs, but CIM performs poorly. No significant differences were observed in CIM results based on the specific GES, OXA-51-like, or ADC β-lactamases identified. CIM-Tris substantially increased detection rates, consistent with previous observations (25).

To the best of our knowledge, this is the first study to comprehensively evaluate and directly compare different CIM-based methods in such a large and diverse collection of GES-β-lactamase producers. A major strength of this work is the size and heterogeneity of the strain collection, which includes several clinically relevant Gram-negative species, and therefore enables a robust assessment of CIM, mCIM, and CIM-Tris across different bacterial genera and GES variants. Gathering this extensive cohort is particularly difficult for GES-type β-lactamases due to detection barriers, making this a breakthrough since no previous study has analyzed a sample size of this magnitude. This collection will be an essential starting point to further expand knowledge on these enzymes, which are of growing concern in healthcare.

The study presented several limitations that warrant consideration. Cut-off values for CIM are not formally established, and in this study, we applied thresholds derived from mCIM and CIM-Tris protocols, which may not represent the optimal cut-offs for this method. A formal assessment of specificity was not performed, as the primary aim of the study was to evaluate the ability of CIM-based methods to detect GES-type carbapenemases. Nevertheless, specificity has been extensively addressed in previous studies (14, 28). In our study, specificity was only assessed in GES-type ESBL isolates, which were exclusively represented by *P. aeruginosa* in our collection. When analyzing the false-positive results reported in this group, we must account for the possibility that these isolates harbor carbapenemases not yet described in genomic databases, especially considering that WGS was utilized as the gold standard. Furthermore, we acknowledged certain limitations regarding our isolate collection. First, the overrepresentation of clonally related *E. cloacae* complex and *P. aeruginosa* isolates may limit the generalizability of our results. We attempted to mitigate this bias by selecting isolates from different patients, across various healthcare facilities, and with broad temporal variation. Additionally, the majority of *S. marcescens* isolates belonged to a single outbreak (26), and most *A. baumannii* isolates co-produced class D carbapenemases, which may also limit the applicability of our findings.

Our results have direct implications for routine clinical microbiology laboratories. In Enterobacterales, particularly the *E. cloacae* complex, GES-type carbapenemases may remain undetected if only a CIM-based method is applied, where a negative result should not rule out the presence of this type of carbapenemase, and it should be confirmed using complementary molecular assays whenever possible. In *P. aeruginosa* GES-carbapenemase producers, mCIM is the best CIM-based method as it provides a good balance between sensitivity and specificity, whereas CIM-Tris should be used with caution due to its low specificity in GES-type ESBL isolates. In *A. baumannii*, phenotypic detection of GES enzymes remains problematic, and molecular confirmation is often required.

In conclusion, CIM-based methods can detect GES-type carbapenemases, but their performance is highly dependent on bacterial species and GES variants. Careful interpretation and complementary molecular testing remain essential.

## Data Availability

All genome sequencing data are available in GenBank under BioProject accession numbers PRJNA1466326, PRJNA1289023, PRJNA1002862, and PRJNA856145.

## SUPPLEMENTAL MATERIAL

Table S1 jcm.00737-26-s0001.docx Collection of isolates included in this study.

ASM does not own the copyrights to Supplemental Material that may be linked to, or accessed through, an article. The authors have granted ASM a non-exclusive, world-wide license to publish the Supplemental Material files. Please contact the corresponding author directly for reuse.
