# Supplementary material for: Evaluation of carbapenem inactivation method-based phenotypic assays for the detection of GES-type carbapenemases in Enterobacterales, Pseudomonas aeruginosa, and Acinetobacter baumannii
Source: J Clin Microbiol. 2026 Aug 21;64(9):e00737-26. doi: 10.1128/jcm.00737-26 (PMC13556193; doi:10.1128/jcm.00737-26)
Supplement: Supplementary file 1 [file jcm.00737-26-s0001.docx]

**SUPPLEMENTAL MATERIAL**

**Table S1.** Collection of isolates included in the study featuring ertapenem MIC, imipenem MIC and meropenem MIC; CIM, mCIM and CIM-Tris results; *bla*GES beta-lactamase variants with their β-lactamic spectrum, carbapenem-producer (CP) condition, co-produced β-lactamases, MLST, isolation year and geographic location.

| **Isolate** | **Microorganism** | **ERT**  **MIC** | **IMI**  **MIC** | **MEM MIC** | **CIM (mm)** | **CIM-Tris (mm)** | **mCIM (mm)** | **GES variant** | **spectrum GES** | **CP** | **Other β-lactamases** | **MLST** | **Year** | **Location** | **Bioproject NCBI** | **Sample number NCBI** |
| --- | --- | --- | --- | --- | --- | --- | --- | --- | --- | --- | --- | --- | --- | --- | --- | --- |
| 1-A1 | *Pseudomonas aeruginosa* | >32 | 32 | >32 | 6 | 6 | 14 | GES-5 | Carbapenemase | Yes | PDC-104, OXA-488 | 235 | 2020 | Spain: Oviedo | PRJNA1466326 | 1-A1 |
| 1-A2 | *Pseudomonas aeruginosa* | >32 | 32 | >32 | 20 | 6 | 16 | GES-5 | Carbapenemase | Yes | PDC-104, OXA-488 | 235 | 2020 | Spain: Oviedo | PRJNA1466326 | 1-A2 |
| 1-A3 | *Pseudomonas aeruginosa* | >32 | 16 | >32 | 6 | 6 | 6 | GES-5 | Carbapenemase | Yes | PDC-104, OXA-488 | 235 | 2020 | Spain: Oviedo | PRJNA1466326 | 1-A3 |
| 1-A4 | *Pseudomonas aeruginosa* | >32 | 32 | >32 | 20 | 6 | 11 | GES-5 | Carbapenemase | Yes | OXA-488, PDC-104 | 235 | 2020 | Spain: Oviedo | PRJNA1466326 | 1-A4 |
| 1-A5 | *Pseudomonas aeruginosa* | 32 | 8 | 4 | 28 | 20 | 22 | GES-15 | ESBL | No | PDC-104, OXA-488 | 235 | 2020 | Spain: Oviedo | PRJNA1466326 | 1-A5 |
| 1-A6 | *Pseudomonas aeruginosa* | >32 | 32 | >32 | 19 | 6 | 8 | GES-5 | Carbapenemase | Yes | PDC-104, OXA-488 | 235 | 2020 | Spain: Oviedo | PRJNA1466326 | 1-A6 |
| 1-A7 | *Pseudomonas aeruginosa* | >32 | 32 | >32 | 6 | 6 | 6 | GES-5 | Carbapenemase | Yes | PDC-104, OXA-488 | 235 | 2021 | Spain: Oviedo | PRJNA1466326 | 1-A7 |
| 1-A8 | *Pseudomonas aeruginosa* | >32 | 32 | >32 | 6 | 6 | 6 | GES-5 | Carbapenemase | Yes | PDC-104, OXA-488 | 235 | 2023 | Spain: Oviedo | PRJNA1466326 | 1-A8 |
| 1-A9 | *Pseudomonas aeruginosa* | >32 | 32 | >32 | 6 | 6 | 6 | GES-5 | Carbapenemase | Yes | OXA-488, PDC-104 | 235 | 2023 | Spain: Oviedo | PRJNA1466326 | 1-A9 |
| 1-B1 | *Pseudomonas aeruginosa* | >32 | 32 | >32 | 20 | 6 | 19 | GES-5 | Carbapenemase | Yes | OXA-488, PDC-104 | 235 | 2024 | Spain: Oviedo | PRJNA1466326 | 1-B1 |
| 1-B3 | *Pseudomonas aeruginosa* | >32 | 32 | >32 | 6 | 6 | 6 | GES-5 | Carbapenemase | Yes | PDC-104, OXA-488 | 235 | 2021 | Spain: Zaragoza | PRJNA1466326 | 1-B3 |
| 1-B4 | *Pseudomonas aeruginosa* | >32 | 8 | 4 | 29 | 17 | 22 | GES-1 | ESBL | No | PDC-104, OXA-488 | 235 | 2023 | Spain: Zaragoza | PRJNA1466326 | 1-B4 |
| 1-B5 | *Pseudomonas aeruginosa* | 16 | 4 | 2 | 27 | 19 | 24 | GES-1 | ESBL | No | PDC-104, OXA-488 | 235 | 2021 | Spain: Madrid | PRJNA1466326 | 1-B5 |
| 1-B6 | *Pseudomonas aeruginosa* | >32 | 32 | >32 | 6 | 6 | 6 | GES-19, GES-20 | Carbapenemase | Yes | OXA-2, PDC-19a, OXA-846 | 309 | 2021 | Spain: Madrid | PRJNA1466326 | 1-B6 |
| 1-B7 | *Pseudomonas aeruginosa* | >32 | 32 | >32 | 6 | 6 | 6 | GES-5 | Carbapenemase | Yes | PDC-104, OXA-488 | 235 | 2022 | Spain: Madrid | PRJNA1466326 | 1-B7 |
| 1-B8 | *Pseudomonas aeruginosa* | >32 | 32 | >32 | 6 | 6 | 6 | GES-5 | Carbapenemase | Yes | PDC-104, OXA-488 | 235 | 2022 | Spain: Madrid | PRJNA1466326 | 1-B8 |
| 1-B9 | *Pseudomonas aeruginosa* | >32 | 32 | >32 | 6 | 6 | 6 | GES-5 | Carbapenemase | Yes | PDC-104, OXA-488 | 235 | 2023 | Spain: Madrid | PRJNA1466326 | 1-B9 |
| 1-C1 | *Pseudomonas aeruginosa* | >32 | 32 | >32 | 6 | 6 | 6 | GES-5 | Carbapenemase | Yes | PDC-104, OXA-488 | 235 | 2023 | Spain: Madrid | PRJNA1466326 | 1-C1 |
| 1-C2 | *Pseudomonas aeruginosa* | >32 | 16 | 32 | 27 | 18 | 24 | GES-1 | ESBL | No | PDC-11, OXA-846 | 357 | 2019 | Spain: Pamplona | PRJNA1466326 | 1-C2 |
| 1-C3 | *Pseudomonas aeruginosa* | >32 | 16 | 32 | 27 | 15 | 22 | GES-1 | ESBL | No | PDC-11, OXA-846 | 357 | 2022 | Spain: Pamplona | PRJNA1466326 | 1-C3 |
| 1-C4 | *Pseudomonas aeruginosa* | >32 | 16 | 8 | 24 | 16 | 21 | GES-1 | ESBL | No | PDC-104, OXA-488 | 235 | 2021 | Spain: Getafe | PRJNA1466326 | 1-C4 |
| 1-C5 | *Pseudomonas aeruginosa* | 32 | 16 | 8 | 27 | 15 | 21 | GES-1 | ESBL | No | PDC-104, OXA-488 | 235 | 2021 | Spain: Getafe | PRJNA1466326 | 1-C5 |
| 1-C6 | *Pseudomonas aeruginosa* | >32 | 32 | >32 | 6 | 6 | 6 | GES-5 | Carbapenemase | Yes | PDC-104, OXA-488 | 235 | 2023 | Spain: Getafe | PRJNA1466326 | 1-C6 |
| 1-D4 | *Pseudomonas aeruginosa* | >32 | 32 | >32 | 6 | 6 | 6 | GES-5 | Carbapenemase | Yes | PDC-104, OXA-488 | 235 | 2022 | Spain: Gran Canaria | PRJNA1466326 | 1-D4 |
| 1-D5 | *Pseudomonas aeruginosa* | >32 | 32 | >32 | 6 | 6 | 6 | GES-5 | Carbapenemase | Yes | OXA-488, PDC-104 | 235 | 2023 | Spain: Gran Canaria | PRJNA1466326 | 1-D5 |
| 1-D6 | *Pseudomonas aeruginosa* | >32 | 32 | >32 | 6 | 6 | 6 | GES-19, GES-20 | Carbapenemase | Yes | OXA-488, PDC-104 | 235 | 2020 | Spain: Madrid | PRJNA1466326 | 1-D6 |
| 1-D7 | *Pseudomonas aeruginosa* | >32 | >32 | 32 | 6 | 6 | 6 | GES-5 | Carbapenemase | Yes | OXA-488, PDC-104 | 235 | 2020 | Spain: Madrid | PRJNA1466326 | 1-D7 |
| 1-D8 | *Pseudomonas aeruginosa* | >32 | 32 | >32 | 6 | 6 | 6 | GES-5 | Carbapenemase | Yes | PDC-104, OXA-488 | 235 | 2020 | Spain: Madrid | PRJNA1466326 | 1-D8 |
| 1-D9 | *Pseudomonas aeruginosa* | >32 | 32 | >32 | 6 | 6 | 6 | GES-5 | Carbapenemase | Yes | OXA-488, PDC-104 | 235 | 2020 | Spain: Madrid | PRJNA1466326 | 1-D9 |
| 1-E1 | *Pseudomonas aeruginosa* | >32 | 32 | >32 | 6 | 6 | 6 | GES-5 | Carbapenemase | Yes | PDC-104, OXA-488 | 235 | 2020 | Spain: Madrid | PRJNA1466326 | 1-E1 |
| 1-E2 | *Pseudomonas aeruginosa* | >32 | 32 | >32 | 6 | 6 | 6 | GES-5 | Carbapenemase | Yes | OXA-488, PDC-104 | 235 | 2020 | Spain: Madrid | PRJNA1466326 | 1-E2 |
| 1-E3 | *Pseudomonas aeruginosa* | >32 | 32 | >32 | 6 | 6 | 6 | GES-5 | Carbapenemase | Yes | PDC-104, OXA-488 | 235 | 2020 | Spain: Madrid | PRJNA1466326 | 1-E3 |
| 1-E4 | *Pseudomonas aeruginosa* | >32 | 32 | >32 | 6 | 6 | 6 | GES-5 | Carbapenemase | Yes | OXA-488, PDC-104 | 235 | 2020 | Spain: Madrid | PRJNA1466326 | 1-E4 |
| 1-E5 | *Pseudomonas aeruginosa* | >32 | 32 | >32 | 6 | 6 | 6 | GES-5 | Carbapenemase | Yes | OXA-488, PDC-104 | 235 | 2020 | Spain: Madrid | PRJNA1466326 | 1-E5 |
| 1-E6 | *Pseudomonas aeruginosa* | >32 | >32 | >32 | 18 | 6 | 6 | GES-19, GES-20 | Carbapenemase | Yes | OXA-488, OXA-2, PDC-104 | 235 | 2020 | Spain: Madrid | PRJNA1466326 | 1-E6 |
| 1-E7 | *Pseudomonas aeruginosa* | >32 | 32 | >32 | 6 | 6 | 6 | GES-5 | Carbapenemase | Yes | OXA-488, PDC-104 | 235 | 2021 | Spain: Madrid | PRJNA1466326 | 1-E7 |
| 1-E8 | *Pseudomonas aeruginosa* | 32 | 16 | 8 | 24 | 18 | 22 | GES-1 | ESBL | No | OXA-488, PDC-104 | 235 | 2021 | Spain: Madrid | PRJNA1466326 | 1-E8 |
| 1-F1 | *Pseudomonas aeruginosa* | >32 | 32 | >32 | 6 | 6 | 6 | GES-5 | Carbapenemase | Yes | PDC-104, OXA-488 | 235 | 2021 | Spain: Madrid | PRJNA1466326 | 1-F1 |
| 1-F3 | *Pseudomonas aeruginosa* | >32 | 32 | >32 | 6 | 6 | 6 | GES-5 | Carbapenemase | Yes | OXA-488, PDC-104 | 235 | 2021 | Spain: Madrid | PRJNA1466326 | 1-F3 |
| 1-F7 | *Pseudomonas aeruginosa* | >32 | 4 | 16 | 28 | 6 | 19 | GES-1 | ESBL | No | OXA-488, PDC-104 | 235 | 2021 | Spain: Madrid | PRJNA1466326 | 1-F7 |
| 1-F8 | *Pseudomonas aeruginosa* | >32 | 32 | >32 | 6 | 6 | 6 | GES-5 | Carbapenemase | Yes | PDC-104, OXA-488 | 235 | 2022 | Spain: Madrid | PRJNA1466326 | 1-F8 |
| 1-F9 | *Pseudomonas aeruginosa* | >32 | 16 | 32 | 25 | 6 | 22 | GES-5 | Carbapenemase | Yes | PDC-104, OXA-488 | 235 | 2022 | Spain: Madrid | PRJNA1466326 | 1-F9 |
| 1-G1 | *Pseudomonas aeruginosa* | >32 | 16 | 32 | 23 | 6 | 19 | GES-5 | Carbapenemase | Yes | PDC-104, OXA-488 | 235 | 2022 | Spain: Madrid | PRJNA1466326 | 1-G1 |
| 1-G2 | *Pseudomonas aeruginosa* | >32 | 16 | 8 | 24 | 14 | 23 | GES-1 | ESBL | No | PDC-104, OXA-488 | 235 | 2022 | Spain: Madrid | PRJNA1466326 | 1-G2 |
| 1-G3 | *Pseudomonas aeruginosa* | 32 | 8 | 8 | 28 | 6 | 23 | GES-1 | ESBL | No | OXA-488, PDC-104 | 235 | 2022 | Spain: Madrid | PRJNA1466326 | 1-G3 |
| 1-G4 | *Pseudomonas aeruginosa* | >32 | 16 | 32 | 27 | 6 | 19 | GES-5 | Carbapenemase | Yes | OXA-488, PDC-104 | 235 | 2022 | Spain: Madrid | PRJNA1466326 | 1-G4 |
| 1-G5 | *Pseudomonas aeruginosa* | >32 | 32 | >32 | 6 | 6 | 6 | GES-5 | Carbapenemase | Yes | PDC-104, OXA-488 | 235 | 2022 | Spain: Madrid | PRJNA1466326 | 1-G5 |
| 1-G6 | *Pseudomonas aeruginosa* | 32 | 8 | 4 | 27 | 20 | 24 | GES-1 | ESBL | No | PDC-104, OXA-488 | 235 | 2022 | Spain: Madrid | PRJNA1466326 | 1-G6 |
| 1-G7 | *Pseudomonas aeruginosa* | >32 | 32 | >32 | 6 | 6 | 6 | GES-5 | Carbapenemase | Yes | PDC-104, OXA-488 | 235 | 2022 | Spain: Madrid | PRJNA1466326 | 1-G7 |
| 1-G8 | *Pseudomonas aeruginosa* | >32 | 32 | >32 | 6 | 6 | 6 | GES-5 | Carbapenemase | Yes | PDC-104, OXA-488 | 235 | 2022 | Spain: Madrid | PRJNA1466326 | 1-G8 |
| 1-G9 | *Pseudomonas aeruginosa* | >32 | 32 | >32 | 6 | 6 | 6 | GES-5 | Carbapenemase | Yes | PDC-104, OXA-488 | 235 | 2023 | Spain: Madrid | PRJNA1466326 | 1-G9 |
| 1-H1 | *Pseudomonas aeruginosa* | >32 | 32 | 32 | 6 | 6 | 6 | GES-5 | Carbapenemase | Yes | PDC-104, OXA-488 | 235 | 2023 | Spain: Madrid | PRJNA1466326 | 1-H1 |
| 1-H3 | *Pseudomonas aeruginosa* | >32 | 32 | >32 | 6 | 6 | 6 | GES-5 | Carbapenemase | Yes | PDC-104, OXA-488 | 235 | 2023 | Spain: Madrid | PRJNA1466326 | 1-H3 |
| 1-H4 | *Pseudomonas aeruginosa* | >32 | 32 | >32 | 6 | 6 | 6 | GES-5 | Carbapenemase | Yes | PDC-104, OXA-488 | 235 | 2023 | Spain: Madrid | PRJNA1466326 | 1-H4 |
| 1-H5 | *Pseudomonas aeruginosa* | >32 | 32 | >32 | 6 | 6 | 6 | GES-5 | Carbapenemase | Yes | PDC-104, OXA-488 | 235 | 2023 | Spain: Madrid | PRJNA1466326 | 1-H5 |
| 1-H6 | *Pseudomonas aeruginosa* | >32 | 32 | >32 | 6 | 6 | 6 | GES-5 | Carbapenemase | Yes | PDC-104, OXA-488 | 235 | 2023 | Spain: Madrid | PRJNA1466326 | 1-H6 |
| 1-H7 | *Pseudomonas aeruginosa* | >32 | 32 | >32 | 6 | 6 | 6 | GES-5 | Carbapenemase | Yes | PDC-104, OXA-488 | 235 | 2023 | Spain: Madrid | PRJNA1466326 | 1-H7 |
| 1-H8 | *Pseudomonas aeruginosa* | >32 | 32 | >32 | 6 | 6 | 6 | GES-5 | Carbapenemase | Yes | PDC-104, OXA-488 | 235 | 2023 | Spain: Madrid | PRJNA1466326 | 1-H8 |
| 1-I2 | *Acinetobacter baumannii* | >32 | 32 | >32 | 15 | 6 | - | GES-11 | ESBL | Yes | OXA-23 | - | 2023 | Spain: Sevilla | - | - |
| 1-I3 | *Pseudomonas aeruginosa* | 32 | 8 | 4 | 25 | 6 | 22 | GES-1 | ESBL | No | PDC-104, OXA-488 | 235 | 2022 | Spain: Madrid | PRJNA1466326 | 1-I3 |
| 1-I4 | *Pseudomonas aeruginosa* | >32 | 32 | >32 | 6 | 6 | 6 | GES-5 | Carbapenemase | Yes | PDC-104, OXA-488 | 235 | 2022 | Spain: Madrid | PRJNA1466326 | 1-I4 |
| 1-I5 | *Pseudomonas aeruginosa* | >32 | >32 | >32 | 6 | 6 | 6 | GES-5 | Carbapenemase | Yes | PDC-104, OXA-488 | 235 | 2023 | Spain: Madrid | PRJNA1466326 | 1-I5 |
| 1-I6 | *Pseudomonas aeruginosa* | >32 | 32 | >32 | 6 | 6 | 6 | GES-5 | Carbapenemase | Yes | PDC-104, OXA-488 | 235 | 2022 | Spain: Madrid | PRJNA1466326 | 1-I6 |
| 1-I7 | *Pseudomonas aeruginosa* | >32 | 32 | >32 | 6 | 6 | 6 | GES-5 | Carbapenemase | Yes | PDC-104, OXA-488 | 235 | 2022 | Spain: Madrid | PRJNA1466326 | 1-I7 |
| 1-I8 | *Pseudomonas aeruginosa* | >32 | 32 | >32 | 6 | 6 | 6 | GES-5, GES-1 | Carbapenemase | Yes | PDC-104, OXA-488 | 235 | 2023 | Spain: Madrid | PRJNA1466326 | 1-I8 |
| 2-A1 | *Pseudomonas aeruginosa* | >32 | 32 | >32 | 6 | 6 | 6 | GES-5 | Carbapenemase | Yes | PDC-104, OXA-488 | 235 | 2024 | Spain: Madrid | PRJNA1466326 | 2-A1 |
| 2-A2 | *Pseudomonas aeruginosa* | >32 | 32 | >32 | 6 | 6 | 6 | GES-5 | Carbapenemase | Yes | PDC-104, OXA-488 | 235 | 2024 | Spain: Madrid | PRJNA1466326 | 2-A2 |
| 2-A3 | *Pseudomonas aeruginosa* | >32 | 4 | 8 | 27 | 19 | 23 | GES-1 | ESBL | No | PDC-104, OXA-488 | 235 | 2024 | Spain: Madrid | PRJNA1466326 | 2-A3 |
| 2-A4 | *Pseudomonas aeruginosa* | >32 | 32 | >32 | 6 | 6 | 6 | GES- | Carbapenemase | Yes | PDC-104, OXA-488 | 235 | 2024 | Spain: Madrid | PRJNA1466326 | 2-A4 |
| 2-A5 | *Pseudomonas aeruginosa* | >32 | 32 | >32 | 6 | 6 | 6 | GES-5 | Carbapenemase | Yes | PDC-104, OXA-488 | 235 | 2024 | Spain: Madrid | PRJNA1466326 | 2-A5 |
| 2-A6 | *Pseudomonas aeruginosa* | >32 | 32 | >32 | 6 | 6 | 6 | GES-5 | Carbapenemase | Yes | PDC-104, OXA-488 | 235 | 2024 | Spain: Madrid | PRJNA1466326 | 2-A6 |
| 2-A7 | *Pseudomonas aeruginosa* | >32 | 16 | >32 | 6 | 6 | 6 | GES-5 | Carbapenemase | Yes | PDC-104, OXA-488 | 235 | 2024 | Spain: Madrid | PRJNA1466326 | 2-A7 |
| 2-A8 | *Pseudomonas aeruginosa* | >32 | 16 | >32 | 21 | 6 | 6 | GES-5 | Carbapenemase | Yes | PDC-104, OXA-488 | 235 | 2022 | Spain: Madrid | PRJNA1466326 | 2-A8 |
| 2-A9 | *Pseudomonas aeruginosa* | >32 | 32 | >32 | 6 | 6 | 6 | GES-5 | Carbapenemase | Yes | PDC-104, OXA-488 | 235 | 2024 | Spain: Madrid | PRJNA1466326 | 2-A9 |
| 2-B1 | *Pseudomonas aeruginosa* | >32 | 32 | >32 | 6 | 6 | 6 | GES-5 | Carbapenemase | Yes | PDC-104, OXA-488 | 235 | 2024 | Spain: Madrid | PRJNA1466326 | 2-B1 |
| 2-B2 | *Klebsiella variicola* | 1 | 2 | 1 | 6 | - | 6 | GES-14 | Carbapenemase | Yes | - | 1582 | 2021 | Spain: Gran Canaria | PRJNA1466326 | 2-B2 |
| 2-B5 | *Pseudomonas aeruginosa* | >32 | 32 | >32 | 6 | 6 | 6 | GES-5 | Carbapenemase | Yes | PDC-104, OXA-488 | 235 | 2024 | Spain: Gran Canaria | PRJNA1466326 | 2-B5 |
| 2-B6 | *Pseudomonas aeruginosa* | >32 | 32 | >32 | 6 | 6 | 6 | GES-5 | Carbapenemase | Yes | PDC-104, OXA-488 | 235 | 2024 | Spain: Gran Canaria | PRJNA1466326 | 2-B6 |
| 2-B7 | *Pseudomonas aeruginosa* | >32 | 32 | >32 | 6 | 6 | 6 | GES-5 | Carbapenemase | Yes | PDC-104, OXA-488 | 235 | 2021 | Spain: Madrid | PRJNA1466326 | 2-B7 |
| 2-B8 | *Pseudomonas aeruginosa* | >32 | 32 | >32 | 6 | 6 | 6 | GES-5 | Carbapenemase | Yes | OXA-488, PDC-104 | 235 | 2021 | Spain: Madrid | PRJNA1466326 | 2-B8 |
| 2-B9 | *Pseudomonas aeruginosa* | >32 | 32 | >32 | 6 | 6 | 6 | GES-5 | Carbapenemase | Yes | OXA-488, PDC-104 | 235 | 2022 | Spain: Madrid | PRJNA1466326 | 2-B9 |
| 2-C1 | *Pseudomonas aeruginosa* | >32 | 32 | >32 | 6 | 6 | 6 | GES-5, GES-1 | Carbapenemase | Yes | PDC-104, OXA-488 | 235 | 2022 | Spain: Madrid | PRJNA1466326 | 2-C1 |
| 2-C2 | *Pseudomonas aeruginosa* | >32 | 16 | >32 | 6 | 6 | 6 | GES-5 | Carbapenemase | Yes | PDC-104, OXA-488 | 235 | 2022 | Spain: Madrid | PRJNA1466326 | 2-C2 |
| 2-C3 | *Pseudomonas aeruginosa* | >32 | 32 | >32 | 6 | 6 | 6 | GES-5 | Carbapenemase | Yes | PDC-104, OXA-488 | 235 | 2022 | Spain: Madrid | PRJNA1466326 | 2-C3 |
| 2-C5 | *Pseudomonas aeruginosa* | >32 | 16 | >32 | 6 | 6 | 6 | GES-5 | Carbapenemase | Yes | PDC-104, OXA-488 | 235 | 2022 | Spain: Madrid | PRJNA1466326 | 2-C5 |
| 2-C6 | *Pseudomonas aeruginosa* | >32 | 32 | >32 | 6 | 6 | 6 | GES-5 | Carbapenemase | Yes | OXA-488, PDC-104 | 235 | 2022 | Spain: Madrid | PRJNA1466326 | 2-C6 |
| 2-C7 | *Pseudomonas aeruginosa* | >32 | 32 | >32 | 6 | 6 | 6 | GES-5 | Carbapenemase | Yes | PDC-104, OXA-488 | 235 | 2023 | Spain: Madrid | PRJNA1466326 | 2-C7 |
| 2-C8 | *Pseudomonas aeruginosa* | >32 | 32 | >32 | 6 | 6 | 6 | GES-5 | Carbapenemase | Yes | OXA-488, PDC-104 | 235 | 2022 | Spain: Madrid | PRJNA1466326 | 2-C8 |
| 2-C9 | *Pseudomonas aeruginosa* | >32 | 32 | >32 | 6 | 6 | 6 | GES-5 | Carbapenemase | Yes | PDC-104, OXA-488 | 235 | 2023 | Spain: Madrid | PRJNA1466326 | 2-C9 |
| 2-D1 | *Pseudomonas aeruginosa* | >32 | 32 | >32 | 6 | 6 | 6 | GES-5 | Carbapenemase | Yes | PDC-104, OXA-488 | 235 | 2023 | Spain: Madrid | PRJNA1466326 | 2-D1 |
| 2-D2 | *Pseudomonas aeruginosa* | >32 | 32 | >32 | 6 | 6 | 6 | GES-5 | Carbapenemase | Yes | PDC-104, OXA-488 | 235 | 2023 | Spain: Madrid | PRJNA1466326 | 2-D2 |
| 2-D3 | *Pseudomonas aeruginosa* | >32 | 32 | >32 | 6 | 6 | 6 | GES-5 | Carbapenemase | Yes | PDC-104, OXA-488 | 235 | 2023 | Spain: Madrid | PRJNA1466326 | 2-D3 |
| 2-D5 | *Pseudomonas aeruginosa* | >32 | 16 | >32 | 6 | 6 | 6 | GES-5 | Carbapenemase | Yes | PDC-104, OXA-488 | 235 | 2023 | Spain: Madrid | PRJNA1466326 | 2-D5 |
| 2-D6 | *Pseudomonas aeruginosa* | >32 | 16 | >32 | 6 | 6 | 6 | GES-5 | Carbapenemase | Yes | PDC-104, OXA-488 | 235 | 2023 | Spain: Madrid | PRJNA1466326 | 2-D6 |
| 2-D7 | *Pseudomonas aeruginosa* | >32 | 32 | >32 | 6 | 6 | 6 | GES-5 | Carbapenemase | Yes | OXA-488, PDC-104 | 235 | 2023 | Spain: Madrid | PRJNA1466326 | 2-D7 |
| 2-D8 | *Pseudomonas aeruginosa* | >32 | 16 | >32 | 6 | 6 | 6 | GES-5 | Carbapenemase | Yes | PDC-104, OXA-488 | 235 | 2023 | Spain: Madrid | PRJNA1466326 | 2-D8 |
| 2-D9 | *Pseudomonas aeruginosa* | >32 | 32 | >32 | 6 | 6 | 6 | GES-5 | Carbapenemase | Yes | PDC-104, OXA-488 | 235 | 2023 | Spain: Madrid | PRJNA1466326 | 2-D9 |
| 2-E1 | *Pseudomonas aeruginosa* | >32 | 32 | >32 | 6 | 6 | 6 | GES-5 | Carbapenemase | Yes | OXA-488, PDC-104 | 235 | 2023 | Spain: Madrid | PRJNA1466326 | 2-E1 |
| 2-E2 | *Pseudomonas aeruginosa* | >32 | 32 | >32 | 6 | 6 | 6 | GES-5 | Carbapenemase | Yes | OXA-488, PDC-104 | 235 | 2024 | Spain: Madrid | PRJNA1466326 | 2-E2 |
| 2-E3 | *Pseudomonas aeruginosa* | >32 | 32 | >32 | 6 | 6 | 6 | GES-5 | Carbapenemase | Yes | OXA-488, PDC-104 | 235 | 2024 | Spain: Madrid | PRJNA1466326 | 2-E3 |
| 2-E4 | *Pseudomonas aeruginosa* | >32 | 16 | >32 | 6 | 6 | 6 | GES-5 | Carbapenemase | Yes | OXA-488, PDC-104 | 235 | 2024 | Spain: Madrid | PRJNA1466326 | 2-E4 |
| 2-E5 | *Pseudomonas aeruginosa* | >32 | 16 | >32 | 6 | 6 | 6 | GES-5 | Carbapenemase | Yes | PDC-104, OXA-488 | 235 | 2024 | Spain: Madrid | PRJNA1466326 | 2-E5 |
| 2-E6 | *Pseudomonas aeruginosa* | >32 | 16 | >32 | 6 | 6 | 6 | GES-5 | Carbapenemase | Yes | PDC-104, OXA-488 | 235 | 2024 | Spain: Madrid | PRJNA1466326 | 2-E6 |
| 2-E7 | *Pseudomonas aeruginosa* | 32 | 8 | 4 | 27 | 19 | 23 | GES-1 | ESBL | No | PDC-104, OXA-488 | 235 | 2023 | Spain: Madrid | PRJNA1466326 | 2-E7 |
| 2-E8 | *Pseudomonas aeruginosa* | 32 | 4 | 4 | 26 | 6 | 24 | GES-1 | ESBL | No | PDC-104, OXA-488 | 235 | 2024 | Spain: Madrid | PRJNA1466326 | 2-E8 |
| 2-E9 | *Pseudomonas aeruginosa* | >32 | 32 | >32 | 6 | 6 | 6 | GES-5 | Carbapenemase | Yes | PDC-104, OXA-488 | 235 | 2024 | Spain: Madrid | PRJNA1466326 | 2-E9 |
| 2-F1 | *Pseudomonas aeruginosa* | >32 | 16 | >32 | 6 | 6 | 6 | GES-5 | Carbapenemase | Yes | OXA-488, PDC-104 | 235 | 2024 | Spain: Madrid | PRJNA1466326 | 2-F1 |
| 2-F2 | *Pseudomonas aeruginosa* | >32 | 16 | >32 | 6 | 6 | 6 | GES-5 | Carbapenemase | Yes | PDC-104, OXA-488 | 235 | 2024 | Spain: Madrid | PRJNA1466326 | 2-F2 |
| 2-F3 | *Pseudomonas aeruginosa* | >32 | 16 | >32 | 6 | 6 | 6 | GES-5 | Carbapenemase | Yes | PDC-104, OXA-488 | 235 | 2024 | Spain: Madrid | PRJNA1466326 | 2-F3 |
| 2-F4 | *Pseudomonas aeruginosa* | >32 | 16 | >32 | 25 | 6 | 6 | GES-5 | Carbapenemase | Yes | PDC-104, OXA-488 | 235 | 2022 | Spain: Madrid | PRJNA1466326 | 2-F4 |
| 2-F5 | *Enterobacter cloacae* complex | 4 | 0.5 | 0.25 | 23 | - | 25 | GES-6 | Carbapenemase | Yes | CTX-M-15 | 66 | 2023 | Spain: Santander | PRJNA1466326 | 2-F5 |
| 2-F6 | *Enterobacter cloacae* complex | 4 | 0.25 | 0.5 | 22 | - | 14 | GES-6 | Carbapenemase | Yes | CTX-M-15 | 66 | 2023 | Spain: Santander | PRJNA1466326 | 2-F6 |
| 2-F9 | *Enterobacter cloacae* complex | 1 | 1 | 0.5 | 6 | - | 6 | GES-6 | Carbapenemase | Yes | CTX-M-15 | 66 | 2019 | Spain: Santander | PRJNA1466326 | 2-F9 |
| 2-G1 | *Enterobacter cloacae* complex | 2 | 0.25 | 0.12 | 25 | - | 13 | GES-6 | Carbapenemase | Yes | CTX-M-15 | 66 | 2019 | Spain: Santander | PRJNA1289023 | 19-2358 |
| 2-G2 | *Enterobacter cloacae* complex | 16 | 1 | 2 | 22 | - | 22 | GES-6 | Carbapenemase | Yes | CTX-M-15 | 66 | 2019 | Spain: Santander | PRJNA1466326 | 2-G2 |
| 2-G6 | *Enterobacter cloacae* complex | 2 | 1 | 1 | 25 | - | 13 | GES-6 | Carbapenemase | Yes | CTX-M-15 | 66 | 2019 | Spain: Santander | PRJNA1289023 | 19-4986 |
| 2-G8 | *Enterobacter cloacae* complex | 4 | 0.5 | 1 | 12 | - | 6 | GES-6 | Carbapenemase | Yes | CTX-M-15 | 66 | 2019 | Spain: Santander | PRJNA1466326 | 2-G8 |
| 2-G9 | *Enterobacter cloacae* complex | 0.5 | 0.5 | 0.12 | 26 | - | 12 | GES-6 | Carbapenemase | Yes | CTX-M-15 | 66 | 2019 | Spain: Santander | PRJNA1466326 | 2-G9 |
| 2-H1 | *Enterobacter cloacae* complex | 1 | 1 | 0.25 | 19 | - | 6 | GES-6 | Carbapenemase | Yes | CTX-M-15 | 66 | 2019 | Spain: Santander | PRJNA1289023 | 19-4474 |
| 2-H3 | *Enterobacter cloacae* complex | 8 | 1 | 1 | 23 | - | 19 | GES-6 | Carbapenemase | Yes | CTX-M-15 | 66 | 2019 | Spain: Santander | PRJNA1466326 | 2-H3 |
| 2-H4 | *Enterobacter cloacae* complex | 4 | 1 | 0.5 | 23 | - | 19 | GES-6 | Carbapenemase | Yes | CTX-M-15 | 66 | 2020 | Spain: Santander | PRJNA1289023 | 20-4146 |
| 2-H5 | *Enterobacter cloacae* complex | 1 | 0.25 | 0.25 | 14 | - | 17 | GES-6 | Carbapenemase | Yes | CTX-M-15 | 66 | 2020 | Spain: Santander | PRJNA1466326 | 2-H5 |
| 2-H6 | *Citrobacter freundii* complex | 0.25 | 0.5 | 0.25 | 6 | - | 6 | GES-6 | Carbapenemase | Yes | CTX-M-15, CMY-150 | 62 | 2020 | Spain: Santander | PRJNA1289023 | 20-5089 |
| 2-H8 | *Enterobacter cloacae* complex | 4 | 1 | 1 | 27 | - | 12 | GES-6 | Carbapenemase | Yes | CTX-M-15 | 66 | 2020 | Spain: Santander | PRJNA1289023 | 20-9267 |
| 2-H9 | *Enterobacter cloacae* complex | 1 | 1 | 0.5 | 22 | - | 6 | GES-6 | Carbapenemase | Yes | CTX-M-15 | 66 | 2020 | Spain: Santander | PRJNA1466326 | 2-H9 |
| 2-I1 | *Klebsiella pneumoniae* | 0.5 | 0.5 | 0.5 | 15 | - | 11 | GES-6 | Carbapenemase | Yes | SHV-108 | 3659 | 2020 | Spain: Santander | PRJNA1289023 | 20-1001 |
| 2-I2 | *Serratia marcescens* | >32 | 4 | 16 | 6 | - | 6 | GES-6 | Carbapenemase | Yes | TEM-12 | - | 2020 | Spain: Santander | - | - |
| 2-I3 | *Enterobacter cloacae* complex | 0.5 | 1 | 0.5 | 25 | - | 16 | GES-6 | Carbapenemase | Yes | CTX-M-15 | 66 | 2020 | Spain: Santander | PRJNA1466326 | 2-I3 |
| 2-I5 | *Enterobacter cloacae* complex | 2 | 2 | 0.5 | 25 | - | 14 | GES-6 | Carbapenemase | Yes | CTX-M-15 | 66 | 2020 | Spain: Santander | PRJNA1466326 | 2-I5 |
| 2-I6 | *Enterobacter cloacae* complex | 1 | 1 | 0.12 | 27 | - | 23 | GES-6 | Carbapenemase | Yes | - | 114 | 2020 | Spain: Santander | PRJNA1466326 | 2-I6 |
| 2-I7 | *Serratia marcescens* | 32 | 4 | 16 | 26 | - | 6 | GES-6 | Carbapenemase | Yes | - | - | 2020 | Spain: Santander | PRJNA1002862 | SM03 |
| 2-I8 | *Serratia marcescens* | 32 | 4 | 16 | 10 | - | 14 | GES-6 | Carbapenemase | Yes | - | - | 2020 | Spain: Santander | PRJNA1002862 | SM04 |
| 2-I9 | *Serratia marcescens* | 32 | 8 | 32 | 6 | - | 6 | GES-6 | Carbapenemase | Yes | - | - | 2020 | Spain: Santander | PRJNA1002862 | SM05 |
| 3-A1 | *Serratia marcescens* | 32 | 8 | 16 | 6 | - | 6 | GES-6 | Carbapenemase | Yes | - | - | 2020 | Spain: Santander | PRJNA1002862 | SM02 |
| 3-A2 | *Serratia marcescens* | >32 | 8 | 16 | 6 | - | 6 | GES-6 | Carbapenemase | Yes | - | - | 2020 | Spain: Santander | PRJNA1002862 | SM01 |
| 3-A3 | *Serratia marcescens* | 32 | 4 | 16 | 6 | - | 6 | GES-6 | Carbapenemase | Yes | - | - | 2020 | Spain: Santander | PRJNA1002862 | SM06 |
| 3-A4 | *Serratia marcescens* | 32 | 8 | 16 | 6 | - | 6 | GES-6 | Carbapenemase | Yes | - | - | 2020 | Spain: Santander | PRJNA1002862 | SM08 |
| 3-A5 | *Serratia marcescens* | 32 | 8 | 16 | 6 | - | 6 | GES-6 | Carbapenemase | Yes | - | - | 2020 | Spain: Santander | PRJNA1002862 | SM07 |
| 3-A6 | *Serratia marcescens* | 16 | 0.5 | 1 | 12 | - | 6 | GES-55 | Carbapenemase | Yes | - | - | 2020 | Spain: Santander | PRJNA1002862 | SM11 |
| 3-A7 | *Serratia marcescens* | 32 | 8 | 16 | 17 | - | 6 | GES-6 | Carbapenemase | Yes | - | - | 2020 | Spain: Santander | PRJNA1002862 | SM13 |
| 3-A8 | *Enterobacter cloacae* complex | 2 | 0.25 | 0.25 | 6 | - | 6 | GES-6 | Carbapenemase | Yes | CTX-M-15 | 66 | 2020 | Spain: Santander | PRJNA1289023 | 20-5402 |
| 3-A9 | *Enterobacter cloacae* complex | 4 | 1 | 0.5 | 23 | - | 17 | GES-6 | Carbapenemase | Yes | MIR-12 | 2416 | 2021 | Spain: Santander | PRJNA1289023 | 21-4145 |
| 3-B1 | *Enterobacter cloacae* complex | 0.5 | 0.5 | 0.25 | 23 | - | 14 | GES-6 | Carbapenemase | Yes | CTX-M-15 | 66 | 2021 | Spain: Santander | PRJNA1466326 | 3-B1 |
| 3-B4 | *Enterobacter cloacae* complex | 4 | 0.5 | 0.5 | 21 | - | 14 | GES-6 | Carbapenemase | Yes | - | 78 | 2021 | Spain: Santander | PRJNA1289023 | 21-6818 |
| 3-B5 | *Enterobacter cloacae* complex | 2 | 0.5 | 0.5 | 25 | - | 20 | GES-6 | Carbapenemase | Yes | CTX-M-15 | 66 | 2021 | Spain: Santander | PRJNA1466326 | 3-B5 |
| 3-B7 | *Enterobacter cloacae* complex | 2 | 0.5 | 0.25 | 21 | - | 22 | GES-6 | Carbapenemase | Yes | CTX-M-15 | 66 | 2021 | Spain: Santander | PRJNA1466326 | 3-B7 |
| 3-B8 | *Citrobacter amalonaticus* | 4 | 1 | 1 | 24 | - | 19 | GES-6 | Carbapenemase | Yes | - | - | 2021 | Spain: Santander | PRJNA1289023 | 21-0291 |
| 3-B9 | *Enterobacter cloacae* complex | 8 | 0.25 | 0.25 | 6 | - | 6 | GES-6 | Carbapenemase | Yes | CTX-M-15 | 66 | 2021 | Spain: Santander | PRJNA1466326 | 3-B9 |
| 3-C2 | *Enterobacter cloacae* complex | 2 | 1 | 0.5 | 6 | - | 6 | GES-6 | Carbapenemase | Yes | CTX-M-15 | 66 | 2022 | Spain: Santander | PRJNA1466326 | 3-C2 |
| 3-C4 | *Enterobacter cloacae* complex | 8 | 1 | 2 | 6 | - | 6 | GES-6 | Carbapenemase | Yes | CTX-M-15 | 66 | 2022 | Spain: Santander | PRJNA1466326 | 3-C4 |
| 3-C5 | *Enterobacter cloacae* complex | 1 | 0.5 | 0.12 | 23 | - | 26 | GES-53 | Carbapenemase | Yes | CTX-M-15 | 66 | 2022 | Spain: Santander | PRJNA1289023 | 22-5198 |
| 3-D5 | *Enterobacter cloacae* complex | 4 | 0.5 | 0.5 | 22 | - | 22 | GES-6 | Carbapenemase | Yes | CTX-M-15 | 66 | 2022 | Spain: Santander | PRJNA1466326 | 3-D5 |
| 3-D7 | *Enterobacter cloacae* complex | 16 | 0.5 | 0.5 | 21 | - | 12 | GES-6 | Carbapenemase | Yes | CTX-M-15 | 66 | 2022 | Spain: Santander | PRJNA1466326 | 3-D7 |
| 3-D9 | *Enterobacter cloacae* complex | 8 | 0.25 | 0.5 | 6 | - | 6 | GES-6 | Carbapenemase | Yes | CTX-M-15 | 66 | 2022 | Spain: Santander | PRJNA1466326 | 3-D9 |
| 3-E2 | *Klebsiella pneumoniae* | 2 | 0.25 | 0.25 | 6 | - | 6 | GES-6 | Carbapenemase | Yes | SHV-187 | 152 | 2022 | Spain: Santander | PRJNA1289023 | 22-5111 |
| 3-E4 | *Enterobacter cloacae* complex | 4 | 1 | 2 | 22 | - | 9 | GES-6 | Carbapenemase | Yes | CTX-M-15 | 66 | 2022 | Spain: Santander | PRJNA1466326 | 3-E4 |
| 3-E5 | *Enterobacter cloacae* complex | 8 | 0.25 | 0.5 | 22 | - | 13 | GES-6 | Carbapenemase | Yes | CTX-M-15 | 66 | 2022 | Spain: Santander | PRJNA1466326 | 3-E5 |
| 3-E6 | *Enterobacter cloacae* complex | 4 | 0.25 | 0.25 | 6 | - | 6 | GES-6 | Carbapenemase | Yes | CTX-M-15 | 66 | 2023 | Spain: Santander | PRJNA1289023 | 23-9747 |
| 3-E7 | *Enterobacter cloacae* complex | 4 | 0.5 | 0.5 | 23 | - | 12 | GES-6 | Carbapenemase | Yes | CTX-M-15 | 66 | 2023 | Spain: Santander | PRJNA1289023 | 23-6402 |
| 3-E8 | *Enterobacter cloacae* complex | 8 | 0.5 | 0.25 | 27 | - | 22 | GES-6 | Carbapenemase | Yes | CTX-M-15 | 66 | 2023 | Spain: Santander | PRJNA1289023 | 23-8802 |
| 3-E9 | *Klebsiella pneumoniae* | >32 | 16 | 32 | 19 | - | 23 | GES-6 | Carbapenemase | Yes | SHV-36 | 253 | 2023 | Spain: Santander | PRJNA1289023 | 23-6350 |
| 3-F1 | *Enterobacter cloacae* complex | 2 | 0.5 | 0.5 | 6 | - | 6 | GES-6 | Carbapenemase | Yes | - | 66 | 2023 | Spain: Santander | PRJNA1289023 | 23-3158 |
| 3-F2 | *Klebsiella michiganensis* | 1 | 0.5 | 0.25 | 6 | - | 6 | GES-6 | Carbapenemase | Yes | OXY-6-1 | 517 | 2023 | Spain: Santander | PRJNA1289023 | 23-9970 |
| 3-F3 | *Enterobacter cloacae* complex | 4 | 0.5 | 0.25 | 23 | - | 24 | GES-6 | Carbapenemase | Yes | CTX-M-15 | 66 | 2023 | Spain: Santander | PRJNA1289023 | 23-3481 |
| 3-F4 | *Enterobacter cloacae* complex | 4 | 0.5 | 0.5 | 21 | - | 23 | GES-6 | Carbapenemase | Yes | CTX-M-15 | 66 | 2023 | Spain: Santander | PRJNA1289023 | 23-5326 |
| 3-F6 | *Enterobacter cloacae* complex | 8 | 1 | 1 | 22 | - | 17 | GES-6 | Carbapenemase | Yes | CTX-M-15 | 66 | 2023 | Spain: Santander | PRJNA1289023 | 23-0011 |
| 3-F7 | *Enterobacter cloacae* complex | 4 | 1 | 1 | 6 | - | 6 | GES-6 | Carbapenemase | Yes | CTX-M-15 | 171 | 2024 | Spain: Santander | PRJNA1466326 | 3-F7 |
| 3-F8 | *Enterobacter cloacae* complex | 4 | 0.5 | 0.5 | 22 | - | 6 | GES-6 | Carbapenemase | Yes | CTX-M-15 | 171 | 2024 | Spain: Santander | PRJNA1466326 | 3-F8 |
| 3-F9 | *Enterobacter cloacae* complex | >32 | 0.5 | 2 | 6 | - | 6 | GES-6 | Carbapenemase | Yes | CTX-M-15 | 171 | 2024 | Spain: Santander | PRJNA1466326 | 3-F9 |
| 3-G1 | *Enterobacter cloacae* complex | 2 | 0.25 | 0.5 | 22 | - | 21 | GES-6 | Carbapenemase | Yes | CTX-M-15 | 66 | 2024 | Spain: Santander | PRJNA1466326 | 3-G1 |
| 3-G2 | *Enterobacter cloacae* complex | 8 | 1 | 2 | 6 | - | 6 | GES-6 | Carbapenemase | Yes | CTX-M-15 | 171 | 2024 | Spain: Santander | PRJNA1466326 | 3-G2 |
| 3-G4 | *Escherichia hermannii* | 0,5 | 0.25 | 0.06 | 25 | - | 6 | GES-6 | Carbapenemase | Yes | - | - | 2024 | Spain: Santander | PRJNA1466326 | 3-G4 |
| 3-G5 | *Acinetobacter baumannii* | 16 | 2 | 2 | 25 | 20 | - | GES-35 | ESBL | No | OXA-65, ADC-117 | 158 | 2020 | Egypt | PRJNA856145 | Ale8 |
| 3-G6 | *Acinetobacter baumannii* | 16 | 2 | 2 | 25 | 6 | - | GES-35 | ESBL | No | OXA-65, ADC-73 | 158 | 2020 | Egypt | PRJNA856145 | Ale12 |
| 3-G7 | *Acinetobacter baumannii* | >32 | 32 | 32 | 23 | 6 | - | GES-11 | ESBL | Yes | OXA-23, OXA-94, ADC-80 | 85 | 2020 | Egypt | PRJNA856145 | Ale16 |
| 3-G8 | *Acinetobacter baumannii* | >32 | 32 | 32 | 19 | 6 | - | GES-11 | ESBL | Yes | OXA-23, OXA-94, ADC-80 | 85 | 2020 | Egypt | PRJNA856145 | Ale18 |
| 3-G9 | *Acinetobacter baumannii* | >32 | 16 | 16 | 19 | 6 | - | GES-11 | ESBL | Yes | OXA-23, OXA-94, ADC-25 | 85 | 2020 | Egypt | PRJNA856145 | Ale19 |
| 3-H1 | *Acinetobacter baumannii* | >32 | 16 | 32 | 19 | 6 | - | GES-35 | ESBL | Yes | OXA-23, OXA-65, ADC117 | 158 | 2020 | Egypt | PRJNA856145 | Ale26 |
| 3-H2 | *Acinetobacter baumannii* | >32 | 16 | 32 | 21 | 6 | - | GES-35 | ESBL | Yes | OXA-23, OXA-65, ADC-117 | 158 | 2021 | Egypt | PRJNA856145 | Ale33 |
| 3-H3 | *Acinetobacter baumannii* | >32 | 16 | 32 | 21 | 6 | - | GES-35 | ESBL | Yes | OXA-23, OXA-65, ADC-117 | 158 | 2021 | Egypt | PRJNA856145 | Ale34 |
| 3-H4 | *Acinetobacter baumannii* | >32 | 16 | 32 | 17 | 6 | - | GES-35 | ESBL | Yes | OXA-23, OXA-65, ADC-117 | 158 | 2021 | Egypt | PRJNA856145 | Ale36 |
| 3-H5 | *Enterobacter cloacae* complex | 0,5 | 0.5 | 0.12 | 25 | - | 23 | GES-6 | Carbapenemase | Yes | CTX-M-15 | 66 | 2024 | Spain: Santander | PRJNA1466326 | 3-H5 |
| 3-H6 | *Enterobacter cloacae* complex | 1 | 0.5 | 0.25 | 15 | - | 24 | GES-6 | Carbapenemase | Yes | CTX-M-15, CTX-M-9 | 66 | 2010 | Spain: Santander | PRJNA1289023 | 10-3174 |
| 3-H8 | *Enterobacter cloacae* complex | 0.5 | 0.5 | 0.12 | 25 | - | 24 | GES-6 | Carbapenemase | Yes | CTX-M-15 | 66 | 2010 | Spain: Santander | PRJNA1289023 | 10-4807 |
| 3-H9 | *Enterobacter cloacae* complex | 0.5 | 0.5 | 0.12 | 28 | - | 25 | GES-6 | Carbapenemase | Yes | CTX-M-15 | 66 | 2010 | Spain: Santander | PRJNA1289023 | 10-5100 |
| 3-I2 | *Enterobacter cloacae* complex | 0.5 | 0.5 | 0.12 | 23 | - | 23 | GES-6 | Carbapenemase | Yes | CTX-M-15 | 66 | 2011 | Spain: Santander | PRJNA1289023 | 11-0386 |
| 3-I3 | *Enterobacter cloacae* complex | 0.5 | 0.5 | 0.25 | 27 | - | 23 | GES-6 | Carbapenemase | Yes | - | 66 | 2011 | Spain: Santander | PRJNA1289023 | 11-1522 |
| 3-I4 | *Enterobacter cloacae* complex | 2 | 0.5 | 0.25 | 22 | - | 22 | GES-6 | Carbapenemase | Yes | - | 66 | 2011 | Spain: Santander | PRJNA1289023 | 11-2874 |
| 3-I5 | *Enterobacter cloacae* complex | 0.25 | 0.5 | 0.12 | 24 | - | 23 | GES-6 | Carbapenemase | Yes | CTX-M-15 | 66 | 2011 | Spain: Santander | PRJNA1289023 | 11-3546 |
| 3-I8 | *Enterobacter cloacae* complex | 1 | 0.5 | 0.12 | 22 | - | 23 | GES-6 | Carbapenemase | Yes | CTX-M-15 | 66 | 2013 | Spain: Santander | PRJNA1289023 | 13-3974 |
| 3-I9 | *Enterobacter cloacae* complex | 2 | 0.5 | 0.25 | 24 | - | 25 | GES-6 | Carbapenemase | Yes | CTX-M-15 | 66 | 2013 | Spain: Santander | PRJNA1289023 | 13-5687 |
| 4-A1 | *Enterobacter cloacae* complex | 4 | 1 | 0.5 | 25 | - | 19 | GES-6 | Carbapenemase | Yes | CTX-M-15 | 66 | 2014 | Spain: Santander | PRJNA1289023 | 14-6222 |
| 4-A2 | *Enterobacter cloacae* complex | 4 | 0.5 | 0.25 | 6 | - | 6 | GES-6 | Carbapenemase | Yes | CTX-M-15 | 66 | 2014 | Spain: Santander | PRJNA1289023 | 14-9095 |
| 4-A3 | *Klebsiella oxytoca* | 1 | 0.25 | 0.12 | 6 | - | 6 | GES-6 | Carbapenemase | Yes | - | 254 | 2014 | Spain: Santander | PRJNA1289023 | 14-3354 |
| 4-A4 | *Enterobacter cloacae* complex | 0.5 | 0.5 | 0.12 | 22 | - | 15 | GES-6 | Carbapenemase | Yes | CTX-M-15 | 66 | 2015 | Spain: Santander | PRJNA1289023 | 15-1075 |
| 4-A6 | *Enterobacter cloacae* complex | 2 | 0.5 | 0.5 | 25 | - | 20 | GES-6 | Carbapenemase | Yes | CTX-M-15 | 66 | 2015 | Spain: Santander | PRJNA1289023 | 15-3705 |
| 4-A7 | *Enterobacter cloacae* complex | 16 | 1 | 1 | 22 | - | 15 | GES-6 | Carbapenemase | Yes | CTX-M-15 | 66 | 2015 | Spain: Santander | PRJNA1289023 | 15-8352 |
| 4-A8 | *Enterobacter cloacae* complex | 2 | 0.5 | 0.25 | 24 | - | 15 | GES-6 | Carbapenemase | Yes | CTX-M-15 | 66 | 2016 | Spain: Santander | PRJNA1289023 | 16-0808 |
| 4-A9 | *Klebsiella oxytoca* | 0.5 | 0.5 | 0.12 | 6 | - | 6 | GES-6 | Carbapenemase | Yes | OXY-2-8 | 2 | 2015 | Spain: Santander | PRJNA1289023 | 15-4388 |
| 4-B1 | *Klebsiella oxytoca* | 1 | 0.5 | 0.5 | 6 | - | 6 | GES-6 | Carbapenemase | Yes | OXY-2-8 | 2 | 2015 | Spain: Santander | PRJNA1289023 | 15-5003 |
| 4-B2 | *Enterobacter cloacae* complex | 16 | 1 | 2 | 25 | - | 18 | GES-6 | Carbapenemase | Yes | CTX-M-15 | 66 | 2016 | Spain: Santander | PRJNA1289023 | 16-3187 |
| 4-B3 | *Enterobacter cloacae* complex | 32 | 0.5 | 0.5 | 25 | - | 20 | GES-6 | Carbapenemase | Yes | - | 66 | 2016 | Spain: Santander | PRJNA1289023 | 16-4041 |
| 4-B4 | *Enterobacter cloacae* complex | 2 | 1 | 0.5 | 22 | - | 16 | GES-6 | Carbapenemase | Yes | CTX-M-15 | 66 | 2016 | Spain: Santander | PRJNA1289023 | 16-4875 |
| 4-B5 | *Enterobacter cloacae* complex | 4 | 0.5 | 0.5 | 24 | - | 20 | GES-6 | Carbapenemase | Yes | - | 66 | 2016 | Spain: Santander | PRJNA1289023 | 16-4922 |
| 4-B6 | *Enterobacter cloacae* complex | 4 | 2 | 1 | 23 | - | 23 | GES-6 | Carbapenemase | Yes | CTX-M-15 | 175 | 2016 | Spain: Santander | PRJNA1289023 | 16-4093 |
| 4-B7 | *Klebsiella oxytoca* | 1 | 0.5 | 0.25 | 6 | - | 6 | GES-6 | Carbapenemase | Yes | OXY-2-8 | 2 | 2016 | Spain: Santander | PRJNA1289023 | 16-4773 |
| 4-B8 | *Enterobacter cloacae* complex | 16 | 0.5 | 2 | 26 | - | 21 | GES-6 | Carbapenemase | Yes | CTX-M-15 | 66 | 2016 | Spain: Santander | PRJNA1289023 | 16-6160 |
| 4-B9 | *Enterobacter cloacae* complex | 8 | 1 | 2 | 20 | - | 17 | GES-6 | Carbapenemase | Yes | CTX-M-15 | 66 | 2016 | Spain: Santander | PRJNA1289023 | 16-6278 |
| 4-C1 | *Klebsiella oxytoca* | 32 | 0.5 | 0.5 | 6 | - | 6 | GES-6 | Carbapenemase | Yes | OXY-2-8 | 2 | 2016 | Spain: Santander | PRJNA1289023 | 16-5678 |
| 4-C2 | *Enterobacter cloacae* complex | 1 | 0.25 | 0.12 | 24 | - | 12 | GES-6 | Carbapenemase | Yes | CTX-M-15 | 66 | 2016 | Spain: Santander | PRJNA1289023 | 16-8899 |
| 4-C3 | *Enterobacter cloacae* complex | 4 | 0.5 | 0.5 | 6 | - | 6 | GES-6 | Carbapenemase | Yes | - | 171 | 2016 | Spain: Santander | PRJNA1289023 | 16-9380 |
| 4-C4 | *Klebsiella oxytoca* | 0.5 | 1 | 0.12 | 6 | - | 6 | GES-6 | Carbapenemase | Yes | OXY-2-8 | 2 | 2016 | Spain: Santander | PRJNA1289023 | 16-7808 |
| 4-C5 | *Enterobacter cloacae* complex | 4 | 0.5 | 0.5 | 6 | - | 6 | GES-6 | Carbapenemase | Yes | - | 171 | 2017 | Spain: Santander | PRJNA1289023 | 17-0543 |
| 4-C6 | *Enterobacter cloacae* complex | 0.12 | 0.5 | 0.12 | 22 | - | 11 | GES-6 | Carbapenemase | Yes | CTX-M-15 | 66 | 2017 | Spain: Santander | PRJNA1289023 | 17-3378 |
| 4-C7 | *Enterobacter cloacae* complex | 0.5 | 0.25 | 0.06 | 25 | - | 22 | GES-6 | Carbapenemase | Yes | CTX-M-15 | 66 | 2017 | Spain: Santander | PRJNA1289023 | 17-5442 |
| 4-C8 | *Enterobacter cloacae* complex | 0.5 | 0.5 | 0.12 | 24 | - | 22 | GES-6 | Carbapenemase | Yes | CTX-M-15 | 66 | 2017 | Spain: Santander | PRJNA1289023 | 17-6365 |
| 4-C9 | *Enterobacter cloacae* complex | 0.25 | 0.5 | 0.06 | 24 | - | 18 | GES-6 | Carbapenemase | Yes | CTX-M-15 | 66 | 2017 | Spain: Santander | PRJNA1289023 | 17-6992 |
| 4-D1 | *Klebsiella oxytoca* | 1 | 0.5 | 0.12 | 6 | - | 6 | GES-6 | Carbapenemase | Yes | OXY-2-8 | 2 | 2017 | Spain: Santander | PRJNA1289023 | 17-5784 |
| 4-D2 | *Enterobacter cloacae* complex | 2 | 1 | 1 | 6 | - | 6 | GES-6 | Carbapenemase | Yes | - | 66 | 2017 | Spain: Santander | PRJNA1289023 | 17-7713 |
| 4-D3 | *Enterobacter cloacae* complex | 8 | 0.5 | 1 | 24 | - | 20 | GES-6 | Carbapenemase | Yes | CTX-M-15 | 66 | 2017 | Spain: Santander | PRJNA1289023 | 17-8231 |
| 4-D4 | *Enterobacter cloacae* complex | 0.5 | 0.5 | 0.06 | 26 | - | 15 | GES-6 | Carbapenemase | Yes | CTX-M-15 | 66 | 2017 | Spain: Santander | PRJNA1289023 | 17-9386 |
| 4-D5 | *Enterobacter cloacae* complex | 0.25 | 0.25 | 0.12 | 25 | - | 19 | GES-6 | Carbapenemase | Yes | CTX-M-15 | 66 | 2018 | Spain: Santander | PRJNA1289023 | 18-2290 |
| 4-D6 | *Klebsiella oxytoca* | 0.5 | 0.5 | 0.12 | 6 | - | 6 | GES-6 | Carbapenemase | Yes | OXY-2-8 | 2 | 2017 | Spain: Santander | PRJNA1289023 | 17-8430 |
| 4-D7 | *Enterobacter cloacae* complex | 2 | 0.5 | 0.5 | 6 | - | 6 | GES-6 | Carbapenemase | Yes | CTX-M-15 | 66 | 2018 | Spain: Santander | PRJNA1289023 | 18-9611 |
| 4-D9 | *Enterobacter cloacae* complex | 0.25 | 0.25 | 0.12 | 26 | - | 12 | GES-6 | Carbapenemase | Yes | CTX-M-15 | 66 | 2024 | Spain: Santander | PRJNA1289023 | 24-5363 |
| 4-E1 | *Klebsiella oxytoca* | 2 | 0.5 | 0.25 | 6 | - | 6 | GES-6 | Carbapenemase | Yes | OXY-2-8 | 2 | 2018 | Spain: Santander | PRJNA1289023 | 18-6681 |
| 4-E2 | *Klebsiella pneumoniae* | 0.25 | 0.25 | 0.12 | 6 | - | 6 | GES-6 | Carbapenemase | Yes | SHV-187 | 251 | 2018 | Spain: Santander | PRJNA1289023 | 18-7550 |
| 4-E3 | *Klebsiella oxytoca* | 1 | 0.25 | 0.06 | 6 | - | 6 | GES-6 | Carbapenemase | Yes | OXY-2-11 | - | 2023 | Spain: Santander | PRJNA1289023 | 23-2388 |
